# Synthesis and Biological Evaluation of an ^18^Fluorine-Labeled COX Inhibitor—[^18^F]Fluorooctyl Fenbufen Amide—For Imaging of Brain Tumors

**DOI:** 10.3390/molecules21030387

**Published:** 2016-03-21

**Authors:** Ying-Cheng Huang, Yu-Chia Chang, Chun-Nan Yeh, Chung-Shan Yu

**Affiliations:** 1 Department of Neurosurgery, Chang-Gung Memorial Hospital at Chiayi and Chang Gung University, Taoyuan 33302, Taiwan; ns3068@gmail.com; 2 Department of Biomedical Engineering and Environmental Sciences, National Tsinghua University, Hsinchu 300, Taiwan; flyinken0221@hotmail.com; 3 Department of Surgery, Chang-Gung Memorial Hospital at Linkou and Chang Gung University, Guei-shan 33305,Taiwan; yehchunnan@gmail.com; 4 Institute of Nuclear Engineering and Science, National Tsing-Hua University, Hsinchu 300, Taiwan

**Keywords:** Inflammation, molecular imaging, NSAIDs, cyclooxygenase

## Abstract

Molecular imaging of brain tumors remains a great challenge, despite the advances made in imaging technology. An anti-inflammatory compound may be a useful tool for this purpose because there is evidence of inflammatory processes in brain tumor micro-environments. Fluorooctylfenbufen amide (FOFA) was prepared from 8-chlorooctanol via treatment with potassium phthalimide, tosylation with Ts_2_O, fluorination with KF under phase transfer catalyzed conditions, deprotection using aqueous hydrazine, and coupling with fenbufen. The corresponding radiofluoro product [^18^F]FOFA, had a final radiochemical yield of 2.81 mCi and was prepared from activated [^18^F]F^−^ (212 mCi) via HPLC purification and concentration. The radiochemical purity was determined to be 99%, and the specific activity was shown to exceed 22 GBq/μmol (EOS) based on decay-corrected calculations. *Ex-vivo* analysis of [^18^F]FOFA in plasma using HPLC showed that the agent had a half-life of 15 min. PET scanning showed significant accumulation of [^18^F]FOFA over tumor loci with reasonable contrast in C6-glioma bearing rats. These results suggest that this molecule is a promising agent for the visualization of brain tumors. Further investigations should focus on tumor micro-environments.

## 1. Introduction

Brain disorders such as Alzheimer’s and Parkinson’s diseases are highly associated with inflammation. Several lines of evidence suggest a critical role for cyclooxygenase type 2 (COX-2) in tumorigenesis [1,2]. It is also known that permanent inactivation of platelet COX-1 restores antitumor reactivity [3,4]. Thus, COX-1 and COX-2 both constitute interesting targets for the development of inhibitors. In contrast to the housekeeping gene COX-1, COX-2 is an inducible enzyme that is expressed at elevated levels at sites of inflammation and malignant transformations [5,6].

COX is a class of enzymes that transforms arachidonic acid (AA) into a series of prostaglandin (PG) analogues. Upon transformation by the aldo-keto reductase superfamily (AKR), the products generated, such as PGs or thromboxane, can induce subsequent inflammation-related signaling pathways. COX enzymes are sequence homodimers composed of tightly associated monomers with identical primary structures. However, COX enzymes function as conformational heterodimers with one allosteric and one catalytic monomer [7]. In addition to catalyzing the transformation of AA, COX-2 also metabolizes endocanabinoids such as donoylethanolamine (AEA) and 2-arachidonoylglycerol (2-AG). The metabolites of these endocanabinoids are analogues of PGs that are involved in the regulation of the arachidonic acid metabolic pathway. COX inhibitors such as traditional non-steroid anti-inflammatory drugs (NSAIDs). COX-2 inhibitors called “coxibs” and both substrate and non-substrate fatty acids have been shown to bind within COX channels [8].

**Figure 1:**
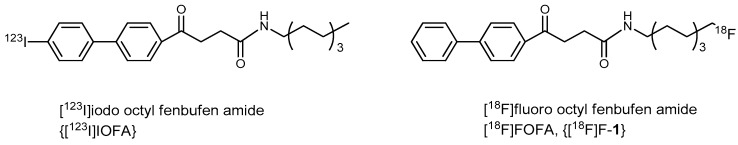
NSAIDs analogue derived radiotracers [^123^I]IOFA and [^18^F]FOFA {[^18^F]F-**1**} studied for SPECT (previous study) and PET imaging (this study) of tumors, respectively.

COX inhibitors are characterized into two main groups: rapid reversible inhibitors, such as ibuprofen and mefenamate, and slow irreversible inhibitors, such as celecoxib, flurbiprofen, and aspirin [9]. A fenbufen analogue, derived from NSAIDs, exhibited cytotoxic effects against cancer cells with an IC_50_ value in the submicromolar range [10,11]. A recent study indicated that 8-[^123^I]iodooctylfenbufen {[^123^I]IOFA} in combination with single photon emission computed tomography (SPECT) could be used to image cholangiocarcinoma in rats [12]. However, imaging of tumors with high mortality rates in clinical settings remains challenging (Figure 1) [13,14,15,16] HPLC analyses of the binding affinities of [^123^I]IOFA to COX enzymes showed a non-selective pattern with a preference for COX-2 over COX-1 (3:2). *In vivo* application of [^123^I]IOFA have been restricted due to its unfavorable aqueous solubility. This physical chemical feature has limited its use in animal studies, as well as in human applications.

To address this issue, the structure was modified by incorporating a fluorine atom (Figure 1). Fluoro groups may form H-bonds with the H_2_O from the aqueous medium facilitating the solvation. Radioactive ^18^F (*t*_1/2_ = 110 min), a positron emitter, is used in positron emission tomography (PET) for molecular imaging purposes and clinical applications [17,18,19]. Due to its better sensitivity and resolution, the use of PET is preferred over the use of SPECT. Its higher sensitivity is particularly valuable for detecting the fewest possible number of cells per unit volume while emitting the least amount of radioactivity. Secondly, the spatial/temporal resolution of PET is significantly higher than that of SPECT and allows for dynamic scanning and the detection of small lesions [20]. Hence, PET combines various chemical probes and offers a tool to investigate biochemical mechanisms *in vivo* [21,22,23].

A number of ^18^F-labeled tracers have been developed and some of these have been demonstrated to be useful for imaging ongoing inflammation [24]. Marnett and co-workers performed a series of sophisticated studies to decipher COX-2 expression and monitor inflammation using ^18^F-coxibs [5]. Encouraged by this advancement, we introduced the hydrogen bond-accepting F atom to improve the hydroaffinity of [^18^F]FOFA for PET applications. This study reports the preparation of [^18^F]FOFA {[^18^F]F-**1**}and assessment of its biological activity *in vitro* and *in vivo*.

## 2. Results and Discussion

**Scheme 1:**
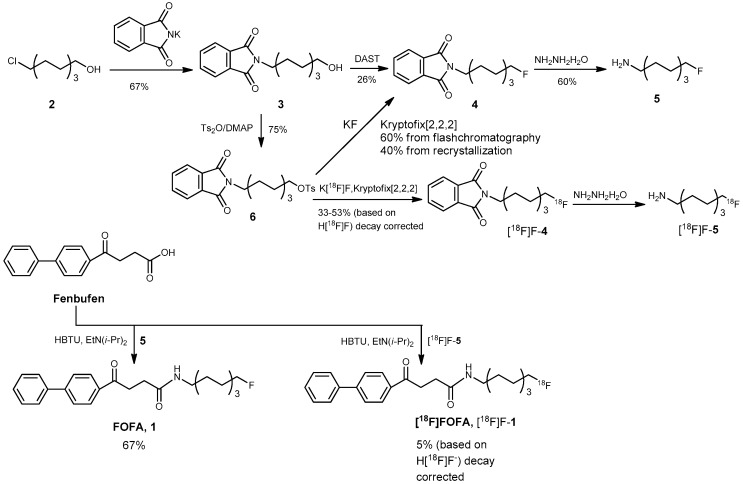
Schematic depiction of the preparation of target [^18^F]FOFA ([^18^F]F-**1**) and the reference non-radioactive standard FOFA (**1**).

Starting with the reaction of 8-chlorooctanol (**2**) with potassium phthalimide via a Gabriel synthesis (Scheme 1), product **5** was formed; however, the fluorination step of compound **3** with diethylamino sulfur trifluoride (DAST) gave an unexpectedly low yield. This lower yield may have been caused by the purity of DAST or the reaction conditions. The yield of the fluorination reaction could be improved by using a different synthetic route that employed a precursor, tosylate **6**, under phase-transfer catalyzed reaction conditions. Hence, with reference compound for FOFA (**1**) in hand, subsequent radiochemical fluorination of tosylate **6** to generate [^18^F]FOFA {[^18^F]F-**1**} was performed.

The purity of precursor **6** was a decisive factor for the radiochemical yield for the radiofluorination reaction. Residual chloride ions can compete with fluoride ions and hamper the radiochemical yields [25]. In the present work, tosylate **6**, prepared from TsCl, did not meet the elemental analysis criteria. This purity concern was resolved by using Ts_2_O as the sources for tosylate moieties. Radiofluorination of **6** was optimized (*n* = 11) using two sources of tosylate coupled with two bases. As shown in Table 1, the radiochemical yield of the fluorination reaction of tosylate **6** obtained using Ts_2_O was obviously higher than that obtained using TsCl.

**Figure 2:**
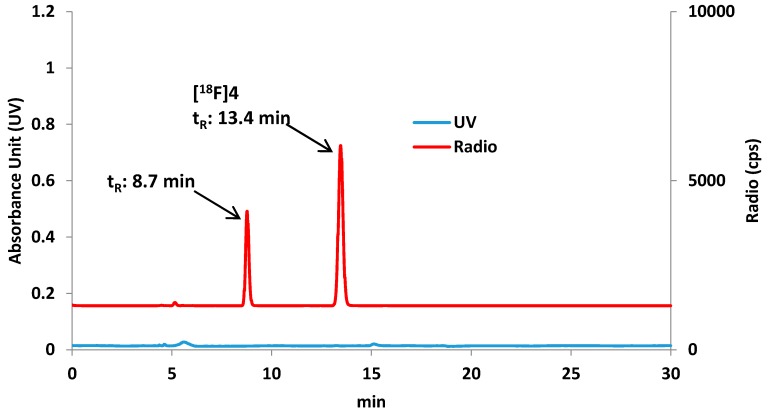
HPLC analysis of the intermediate mixture of [^18^F]F**-4**; unknown at *t*_R_ = 8.7 min is suggested to be an eliminated alkene product as shown below. RCY of eliminated product was *ca*. 15%. ZORBAX SIL 5 μm 9.4 × 250 mm, EtOAc:*n*-hexane = 1:2, 3 mL/min.

**Scheme 2:**
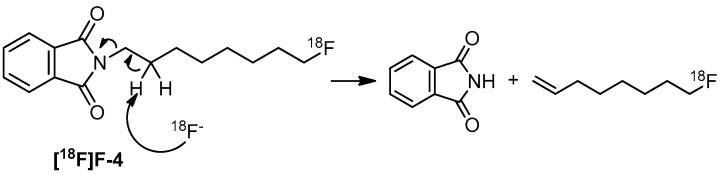
Proposed eliminated product derived from [^18^F]**4**.

Radiofluorination of tosylate **6** afforded [^18^F]F**-4** with a radiochemical yield of 53%, as determined by an HPLC chromatogram (decay corrected, Figure 2). The probable mechanism for the appearance of an unknown compound (*t*_R_ = 8.7 min) was illustrated in Scheme 2. Subsequent restoration of the amino groups was accomplished using aqueous hydrazine. However, after several purification trials using either normal phase or reverse phase HPLC under various elution conditions, we failed to generate a satisfactory HPLC chromatogram. Thus, purification was performed after the subsequent amide-formation step. Starting from the radioactivity of activated [^18^F]F^−^ (212 mCi) obtained by repeated distillation with Kryptofix[2,2,2], a three-hour manipulation that included radiofluorination, deprotection, amide formation, HPLC purification, drying and finally dissolving the reaction product in aqueous 20% EtOH for subcutaneous injection afforded the target compound [^18^F]FOFA {[^18^F]-**1**} in 4% radiochemical yield (2.81 mCi). The final product had a radiochemical purity of 99% and specific activity of 22 GBq/μmol (EOS), based on decay-corrected calculations (*n* = 3). The identity of [^18^F]FOFA {[^18^F]**-1**} was confirmed by HPLC analysis in the presence of the nonradioactive FOFA (**1**) reference compound (Supplementary Materials, Figure S1). Cold synthesis using the same procedure through column chromatography generated the desired product FOFA (**1**) in sufficient purity. No specific impurities with similar *t*_R_s was observed in UV chromatogram of [^18^F]FOFA {[^18^F]-**1**} because the polarities between the precursor, fenbufen, and the product FOFA (**1**), were significantly different.

**Figure 3:**
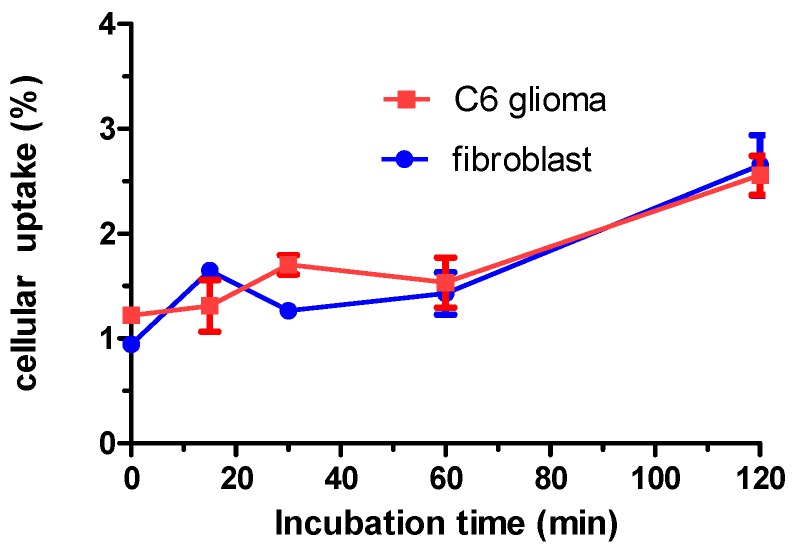
Comparison of internalization rate of [^18^F]FOFA {[^18^F]**-1**} between C6-glioma brain tumour and the control fibroblast cells. Increasing uptake rate is remarkable in the lateral stage. Data are from one experiment with triplicate samples and are expressed as mean ± SD.

Our previous report of an analogue of [^18^F]FOFA {[^18^F]**-1**}, [^123^I]IOFA, showed comparable binding affinities to COX-1 and COX-2 enzymes in micromolarities. Due to their structural similarities, the present work of [^18^F]FOFA {[^18^F]-**1**} focused on assessing its aqueous solubility and bioavailability. Its radiotracer cell uptake increased with prolonged incubation times (Figure 3). In contrast, cellular uptake of the tracer showed marginal selectivity, and image contrast could be optimized if the clearance rate was sufficiently rapid.

**Figure 4:**
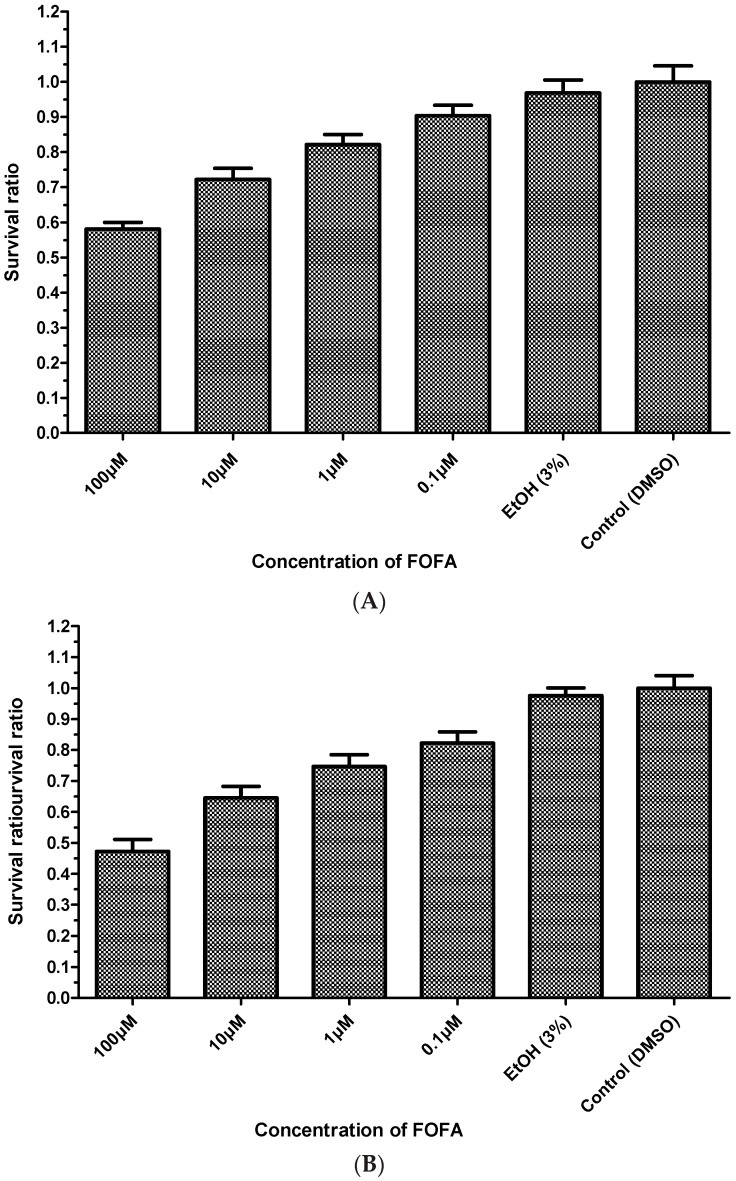
Cell survival assay of FOFA {[^18^F]-**1**} in (**A**) fibroblast (a control) and (**B**) C6 glioma tumour cells after 48 h.

As shown in Figure 4, a cell viability test of FOFA **1** in both C6 glioma and fibroblast cells showed comparable inhibitory activities, but C6 glioma cells exhibited increased cytotoxicity in (IC_50_ < 100 uM). Together, these features may be indicative of its application *in vivo*.

**Figure 5:**
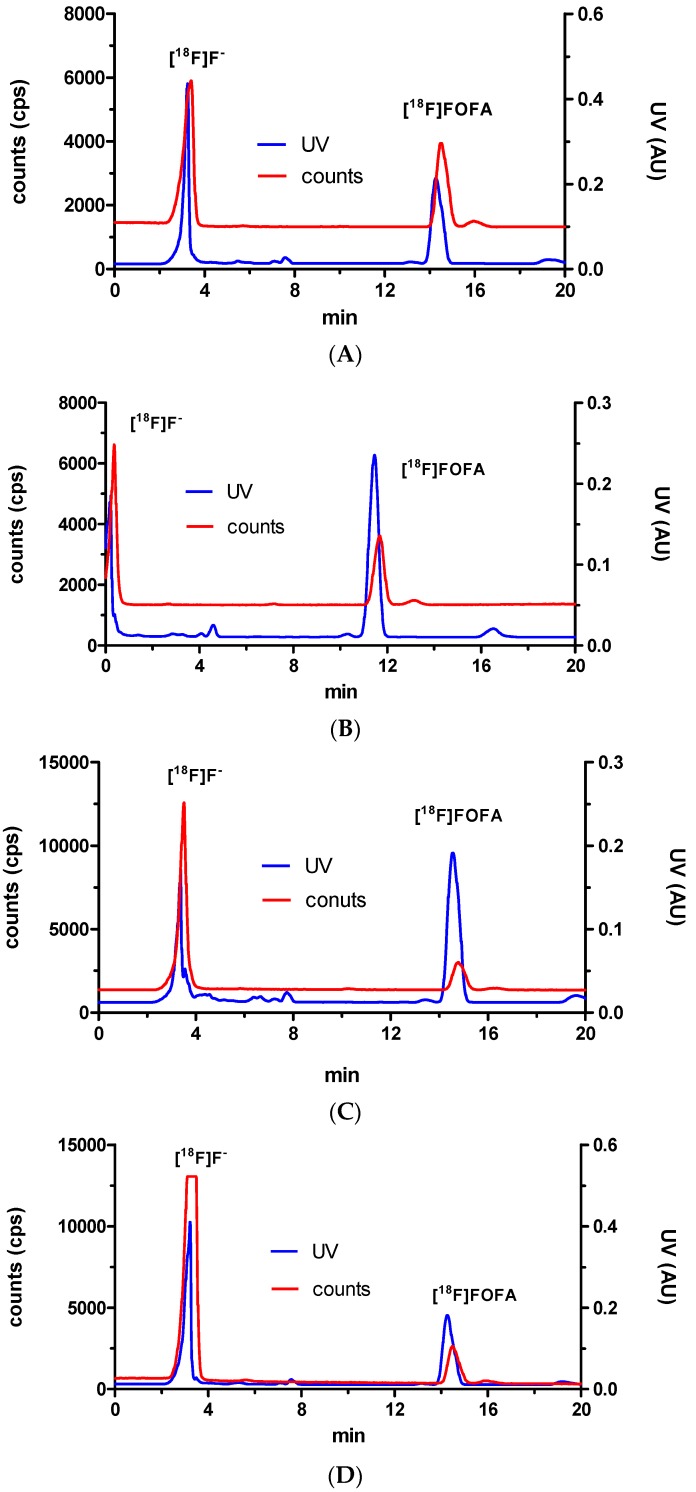
HPLC profiles of the soluble fraction of the [^18^F]FOFA {[^18^F]F**-1**} mixture in plasma at different times post injection, λ_abs_ = 260 nm. For comparison, a small amount of authentic nonradioactive FOFA **1** was added to the mixture as a referential ultraviolet signal. *t*_R_ = 3.41 min, [^18^F]F^−^; *t*_R_ = 14.51 min, [^18^F]FOFA {[^18^F]F-**1**}. Samples were taken from mixture after 10 s (**A**); 2 min (**B**); 10 min (**C**) and 30 min (**D**). The time delay of 3 min in the chromatogram (**B**) was denoted due to an unexpected status.

**Figure 6:**
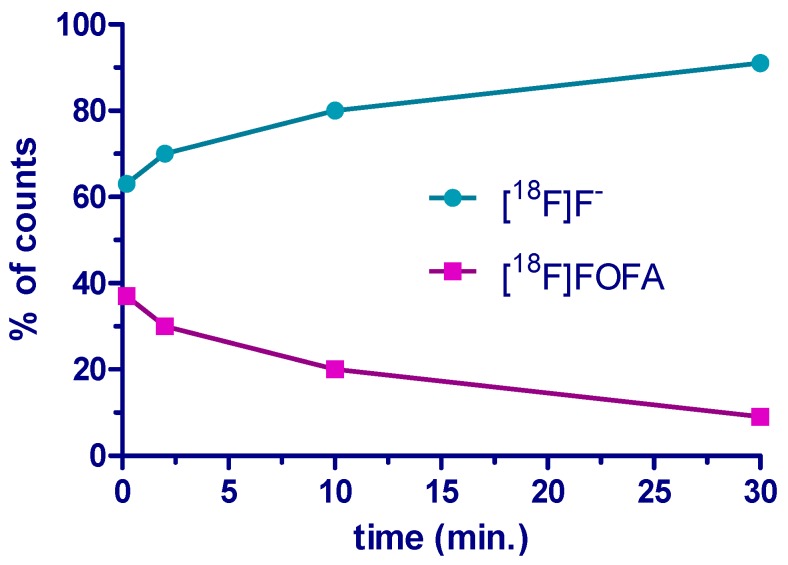
Illustration of [^18^F]FOFA {[^18^F]F-**1**}stability in terms of time activity curve by observing the decline of [^18^F]FOFA {[^18^F]F-**1**}and growth of the metabolites [^18^F]F^−^ from peak integration in HPLC chromatogram. The *ex vivo* half-life is approximately *t*_1/2_ < 1 min.

To assess the *in vivo* stability of [^18^F]FOFA, an alternative environment containing a flask of plasma was used as an *ex vivo* model. The *ex vivo* test was performed by first mixing the tracer with plasma samples for specific time intervals, *i.e.*, 10 s, 2 min, 10 min and 30 min. After injecting the soluble fractions of the plasma mixture of [^18^F]FOFA {[^18^F]F-**1**} into an HPLC system, the intact [^18^F]FOFA {[^18^F]F-**1**} and the released [^18^F]F^−^ were observed concomitantly in the chromatogram (Figure 5). The two species were further illustrated using their time activity profiles, as shown in Figure 6. Its short half life of less than 1 min implied its instability.

**Figure 7:**
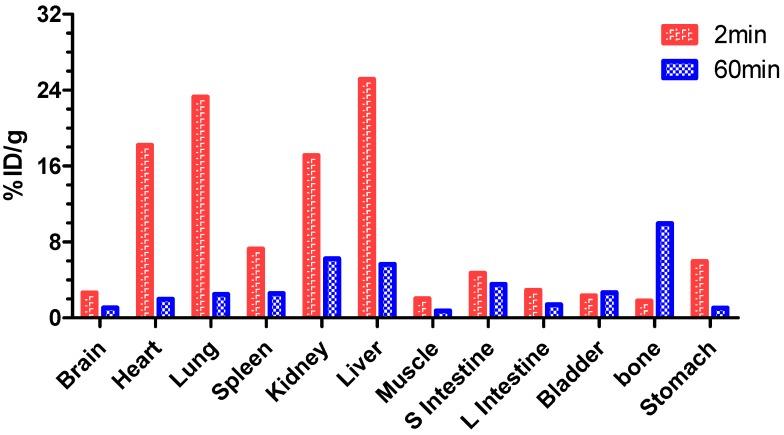
Biodistribution of [^18^F]FOFA {[^18^F]F-**1**} in healthy male SD rats. The two rats were injected intravenously with 0.1 mCi and were euthanized and dissected after injection at 2 min and at 60 min, respectively. Data are presented as %ID/g. The measurement was performed in triplicate.

As expected, 20% EtOH (aq) was capable of dissolving [^18^F]FOFA {[^18^F]F-**1**} during injection preparation for animal PET study. This impressive solubility reminded us the contradictory experience of observing a lot of suspended particles with [^123^I]IOFA. Additionally, a biodistribution experiment was performed in two normal rats (Figure 7). The results showed significant accumulation in bone tissues at 60 min post-injection. Approximately 1%–3% ID/g of activity was distributed over the brain. The relatively nonpolar feature of FOFA {[^18^F]F-**1**} did not impact its penetration across the blood brain barrier prior to defluorination because the significant activity observed in bone tissues was denoted 60 min after injection. These results were consistent with the findings from the HPLC analyses.

The *in vitro* stability test using plasma indicated that a significant amount of [^18^F]F-**1** was released at 2 min post-injection. However, the radiotracer had not significantly accumulated in the bone tissue at 2-min resection from the *in vivo* test. This may have been caused by short observation times which may have been insufficient for reaching an equilibrium within 2 min.

**Figure 8:**
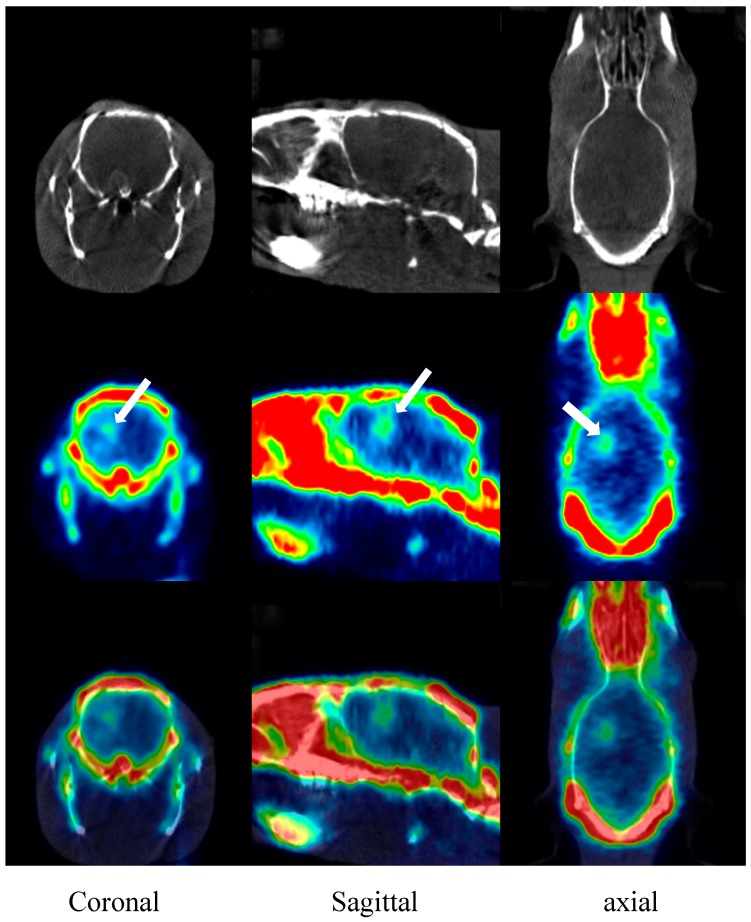
A static PET/CT image collected over a period of 0 to 60 min of [^18^F]FOFA {[[^18^F]F-**1**} in the C6 glioma-bearing rat with an injection dose of 2.81 mCi/1 mL of [^18^F]FOFA{[^18^F]F-**1**}. Organs scanned are shown for the head part. The first row shows CT images; the second row shows PET images; the third shows their overlay.

To assess the molecular imaging applicability, a rat that was inoculated with a tumor on the right hemisphere of the brain was injected with a dose of 2.81 mCi/1 mL via a tail-vein injection. The tumor loci was easily identified by MRI before PET scanning, as reported previously [26,27]. The animal was assessed using static PET imaging over a 60-min time frame as indicated in Figure 8. The results showed that most of the radioactivity was accumulated in the bone region. An obvious accumulation over the tumor region with reasonable contrast from the background was also noted at 60 min post-injection.

**Scheme 3:**
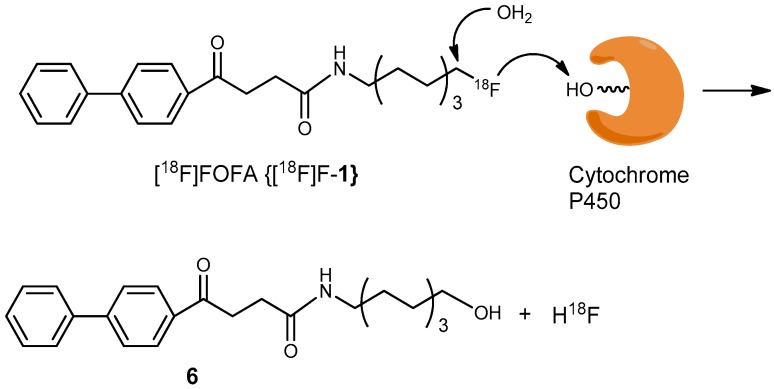
The mechanism for removal of fluoride from [^18^F]FOFA{[^18^F]F-**1**} *in vivo* was proposed by lipase-catalysed hydrolysis through proton donation from the crboxyl or thiol group from amino acid side chains e.g., aspartate, glutamate or cysteine.

Imaging data indicated that the brain tumor absorbed higher amount of the radiotracer to maintain COX metabolism. Regions where COX metabolism is active enough to be traced by PET at early stages of tumor progression require a more stable radiotracer. Although C-F bonding is strong enough to resist bond cleavage [28], catalytic protonation of poor leaving groups could readily transform F^−^ to HF, which is a good leaving group. A hepatic enzyme with a housekeeping role such as cytochrome P450, may be able to address this mechanism (Scheme 3) [29]. Carbonyl and acyl halides have been suggested to form after initial hydroxylation at the α-position of carbon–fluorine bonds promoting the subsequent removal of hydrogen fluoride [28,30].

In brief, the present work demonstrates the preparation of an octylfenbufen analogue by modifying the terminal end of the octyl group with a fluorine atom for use as a PET tumor-imaging probe for tumors and for inflammation processing. Modifying the terminal end group was shown to improve the solubility of the compound in a 20% EtOH aqueous solution according to the observation of injection preparation before PET scanning.

## 3. Materials and Methods

### 3.1. General Information

All reagents and solvents were purchased from Sigma-Aldrich (St. Louis, MO, USA), Fluka (St. Louis, MO, USA), Acros (Geal, Belgium), Alfa (Binfield, Berkshire, UK), Malingkrodt (Phillipsburg, NJ, USA), or Tedia (Farfield, OH, USA). Preparation of all nonradioactive compounds, except for compound **5**, was routinely conducted in dried glassware under a positive pressure of nitrogen at room temperature unless otherwise noted. CH_2_Cl_2_, toluene, CH_3_CN, and pyridine were dried over CaH_2_ and CH_3_OH was dried over Mg and distilled prior to their use in reactions. Solvents (e.g., DMF and N(*i*-Pr)_2_Et) were distilled under reduced pressure. All the reagents and solvents were of reagent grade. Dimethylaminopyridine (DMAP) was purified by recrystallizing the reagent from a combination of EtOAc and *n*-hexane before use. The eluents for flash chromatography (e.g., EtOAc, acetone, and *n*-hexane) were of industrial grade and were distilled prior to use; CH_3_OH and CHCl_3_ were of reagent grade and used without further purification. Nylone filters with 0.45 μM pore size were supplied by Waters (Nilford, MA, USA). NMR spectroscopy including ^1^H-NMR (500 MHz),^13^C-NMR (125 MHz, DEPT-135) and ^19^F-NMR (470 or 564 MHz) was performed on a Unity Inova 500 MHz instrument (Varian, USA). Deuterated-solvents employed for NMR spectroscopy including CD_3_OD, CDCl_3_, C_6_D_6_, and DMSO-*d*_6_ were purchased from Aldrich. Low-Resolution Mass Spectrometry (LRMS) was performed on an ESI-MS spectrometer using a Varian 901-MS Liquid Chromatography Tandem Mass Q-TOF Spectrometer at the Department of Chemistry of National Tsing-Hua University (NTHU) or the Department of Applied Chemistry of National Chio-Tung University (NCTU). High-Resolution Mass Spectrometry (HRMS) was performed using a Varian HPLC (prostar series ESI/APCI) system coupled with a Varian 901-MS (FT-ICR Mass) mass detector and a triple quadrapole instrument. Elemental analyses were performed using an Elemental Analysis, CHN-O-RAPID apparatus (Foss Heraeus, Burladingen, Germany). Thin layer chromatography (TLC) was performed with TLC silica gel 60 F_254_ pre-coated plates (Machery-Nagel, Dueren, Germany) to monitor the starting materials and products with visualization under UV light (254 nm). Further confirmation was carried out by staining the TLC plates with 5% *p*-anisaldehyde, ninhydrin or ceric ammonium molybdate under heating. Celite 545 was purchased from Macherey-Nagel Inc. (Dueren, Germany). Flash chromatography was performed using Silicycle 60 silica gel (70–230 mesh, Quebec City, QC, Canada). Melting points were measured with a MEL-TEMP instrument (Barnstead International, Dubuque, IA, USA) and were uncorrected.

[^18^F]HF was produced on a GE PET tracer cyclotron (Milwaukee, WI, USA) by an ^18^O(p,n) nuclear reaction (NERI, Longtan, 32546, Taiwan). The radiolabeling experiment was performed on a GE TracerLAB FX_FN_ synthesis module (GE Medical Systems, Milwaukee, WI, USA). PET imaging was performed with a NanoPET/CT (MEDISO Inc., Knoxville, TN, USA) instrument at the Nuclear Energy Research Institute. The intermediate product, [^18^F]F**-4**, was analyzed with an Waters HPLC system (Milford, MA, USA) consisting of a Waters 510 pump, a linear UVIS detector (254 nm) in series with a Berthhold (Bad Wildbad, Baden Wuetermberg, Germany) γ-flow detector and a ZORBAX SILcolumn (9.4 mm × 250 mm, 5 μm) using isocratic EtOAc/*n*-hexane 1:2 as the mobile phase with a flow rate of 3 mL/min. The mixture of [^18^F]FOFA {[^18^F]F**-1**} obtained from the FX_FN_ synthesis module (TracerLAB, Amersham, UK) was purified with the same HPLC settings as described above; however, a reverse phase Develosil ODS-7 (5 μm 10 × 250 mm) column and a mobile phase consisting of H_2_O–CH_3_CN = 30:70 in isocratic mode was utilized for these HPLC analyses. The identity of the radiolabeled compound was confirmed by co-injecting it with an authentic standard into an HPLC for analysis. The area under the UV absorbance peak measured at 260 nm, which corresponded to the carrier product, was calculated and compared to a standard curve to relate mass to UV absorbance. Only specific activity below 40 GBq/μmol could be measured accurately. Radioactivity was measured using a R15C dose calibrator (Capintec, Ramsey, NJ, USA). *Ex-vivo* stability studies were performed on the system as described, but using a CHEMCOSORB 7-ODS-H column (10 × 250 mm, 5 μm) and an eluent system that had the following gradient program: CH_3_CN/0.05% trifluoracetic acid = 20/80 at 0 min to CH_3_CN /0.05% trifluoracetic acid = 95/5 at 10 min and a further gradient to CH_3_CN (100%) at 20 min.

### 3.2. Chemical Syntheses

#### *2-(8-Hydroxyoctyl)isoindoline-1,3-dione* (**3**)

A mixture of ClCH_2_(CH_2_)_7_OH (**2**, 4 mL, 23.7 mmol) and potassium phthalimide (5.26 g, 1.2 eq, 28.4 mmol) in dimethyl formamide (DMF, 12 mL) was stirred under reflux (160 °C) for 2 h. TLC analysis (EtOAc–*n*-hexane = 4:6) indicated the consumption of starting material **2** (R*_f_* = 0.48) and the formation of product **3** (R*_f_* = 0.3). The mixture was then transferred to a separation funnel for an extraction with CH_2_Cl_2_ (130 mL) and 1N HCl (50 mL × 2). The organic layer was washed with saturated NaHCO_3_ (aq) followed by drying with Na_2_SO_4_. After filtration through a pad of celite, the filtrate was sequentially concentrated using a membrane pump and an oil pump, under reduced pressure at 50 °C. A brown viscous oil was obtained and was purified using flash chromatography under the following conditions: a column that was 6 cm in diameter and using 250 g of silica gel and EtOAc–*n*-hexane = 4:6 eluent system. The product **3** was obtained in 70% yield (4.5 g). After recrystallization from CH_3_OH, a colorless, slightly white crystal was obtained in 67% yield (4.41 g). m.p. 63–64 °C (60-62 °C, 42% yield; [31] white wax, 72%) [32]. Calcd. C_16_H_21_NO_3_ MW = 275.3, ESI + Q-TOF MS, M = 275.2 (*m*/*z*), [M + Na]**^+^** = 298.1; HRMS-ESI, Calcd. [M + H]^+^ = 276.1600, [M + Na]^+^ = 298.1419; found: [M + H]^+^ = 276.1597, [M + Na]^+^ = 298.1419; Elemental analysis: Calcd. C, 69.79; H, 7.69; N, 5.09. Found C, 69.46; H, 7.81; N, 5.36. ^1^H-NMR (500 MHz, CDCl_3_): δ 1.32–1.33 (m, 8H, CH_2_), 1.52–1.56 (m, 2H, CH_2_), 1.65–1.69 (m, 2H, CH_2_), 2.16 (s, 1H, OH), 3.62 (t, *J* = 6.5 Hz, 2H, H-8), 3.67 (t, *J* = 7.5 Hz, 2H, H-1 ), 7.68–7.72 (m, 2H, Ar), 7.81–7.85 (m, 2H, Ar); ^13^C-NMR (125 MHz, CDCl_3_): δ 25.58 (CH_2_), 26.70 (CH_2_), 28.50 (CH_2_), 29.04 (CH_2_), 29.18 (CH_2_), 32.67 (CH_2_), 37.98 (CH_2_), 62.96 (CH_2_), 123.13 (CH, arom), 132.12 (C, arom), 133.82 (CH, arom), 168.47 (C, CO_amide_).

#### *8-(1,3-Dioxoisoindolin-2-yl)octyl 4-methylbenzenesulfonate* (**6**)

Bartholomä *et al.* [32] utilized TsCl to prepare compound **6**. Dimethylaminopyridine (DMAP, 181 mg, 1.48 mmol, 1.9 eq) was dried by co-distillation with toluene (1 mL) thrice. Compound **3** was also co-distilled with toluene (1 mL) thrice and was dissolved in CH_2_Cl_2_ (3 mL) in a two-necked round bottom flask. DMAP was then added to this solution and the mixture was stirred in an ice bath. Then, toluene sulfonic anhydride (Ts_2_O, 473 mg, 1.45 mmol, 1.9 eq) was added and the stirring continued for 30 min. After removal of the ice bath, the reaction was allowed to continue at RT for 2 h. A TLC (EtOAc–*n*-hexane = 4:6) analysis indicated the consumption of starting material **3** (R*_f_* = 0.25) and the formation of product **6** (R*_f_* = 0.55). The mixture was then partitioned between 1N HCl (30 mL) and CH_2_Cl_2_ (60 mL) in a separation funnel. The organic layer was dried with Na_2_SO_4_ followed by filtration through a celite pad. The filtrate was concentrated under reduced pressure to give dark brown colored viscous oil (310 mg). The residue was purified by flash chromatography in a column that was 2 cm in diameter with 20 g silica gel using EtOAc–*n*-hexane = 2:8 as an eluent to afford the product **6** as a pale yellow oil in 75% yield (246 mg). The product synthesized by Bartholomä *et al.* was obtained as a colorless oil in 45% yield. and elemental analysis data were not available. Preparation of the compound using toluene sulfonyl chloride (TsCl) did not provide product **6** with satisfactory purity according to the elemental analysis: H, 5.82 (6.34, calcd.). Calcd. C_23_H_27_NO_5_S, MW = 429.5, ESI + Q-TOF MS, M = 429.2 (*m*/*z*), [M + H]^+^ = 430.1 (100%), [M + Na]^+^ = 452.1, [2M + Na]^+^ = 881.5; HRMS-ESI, Calcd. [M + H]^+^ = 430.1688, [M + Na]^+^ = 452.1508; found: [M + H]^+^ = 430.1688, [M+Na]^+^ = 452.1506; Elemental analysis: Calcd. C, 64.31; H, 6.34; N, 3.26. Found C, 64.32; H, 6.33; N, 3.40. ^1^H-NMR (500 MHz, CDCl_3_): δ 1.20–1.30 (m, 8H, CH_2_), 1.58–1.65 (m, 4H, CH_2_), 2.44 (s, 3H, CH_3_), 3.65 (t, *J* = 7.0 Hz, 2H, H-1), 4.00 (t, *J* = 6.5 Hz, 2H, H-8), 7.34 (d, *J* = 8.5 Hz, 2H, Ar), 7.70–7.72 (m, 2H, Ar), 7.78 (d, *J* = 8.0 Hz, 2H, Ar), 7.82–7.84 (m, 2H, Ar); ^13^C-NMR (125 MHz, CDCl_3_): δ 21.59 (CH_3_), 25.20 (CH_2_), 26.63 (CH_2_), 28.46 (CH_2_), 28.71 (CH_2_), 28.73 (CH_2_), 28.86 (CH_2_), 37.91 (CH_2_), 70.57 (CH_2_), 123.13 (CH, arom), 127.85 (CH, arom), 129.77 (CH, arom), 132.12 (C, arom), 133.21 (C, arom), 133.84 (CH, arom), 144.59 (C, arom), 168.42 (C, CO_amide_).

#### *2-(8-Fluorooctyl)isoindoline-1,3-dione* (**4**)

##### Route 1 *via fluorination with diethyl aminosulfur trifluoride (DAST)*

A similar product with a truncated heptyl chain was reported by Li *et al.* [33]. A mixture of compound **3** (2 g, 7.3 mmol) in CH_2_Cl_2_ (5 mL) was stirred at −40 °C. DAST (1.6 mL, 13 mmol, 1.8 eq) was added portionwise within 20 s, and the reaction was stirred for 2 h. TLC (EtOAc–*n*-hexane = 4:6) analysis indicated consumption of the starting material **3** (R*_f_* = 0.28) and formation of the product **4** (R*_f_* = 0.65). The mixture was then partitioned between CH_2_Cl_2_ (60 mL) and NaHCO_3(aq)_ (30 mL × 2) in a separation funnel. The organic layer was dried using Na_2_SO_4_ followed by filtration through a celite pad. The filtrate was concentrated under reduced pressure to give a yellow solid (650 mg). This crude compound was purified by flash chromatography in a column that was 3 cm in diameter with 50 g of silica gel using a mixture of EtOAc–*n*-hexane = 1:9 as an eluent, product **4** was obtained as a white solid in 27% yield (550 mg). After recrystallization from CH_3_OH, a colorless crystal was obtained in 26% yield (523 mg). m.p. 51–52 °C. Calcd. C_16_H_20_FNO_2_, MW = 277.3, ESI + Q-TOF MS, M = 277.1 (*m*/*z*), [M]^+^ = 277.1, [M + Na]^+^ = 300.1, [M + K]^+^ = 316.1; HRMS-ESI , Calcd. M = 277.1418 (*m*/*z*), [M + H]^+^ = 278.1556, [M + Na]^+^ = 300.1376, [M + K]^+^ = 316.1115; found: [M + H]^+^ = 278.1543, [M + Na]^+^ = 300.1363, [M + K]^+^ = 316.1284; Elemental analysis: Calcd. C, 69.29; H, 7.27; N, 5.05. Found C, 69.19; H, 7.63; N, 5.35. ^1^H-NMR (500 MHz, CDCl_3_): δ 1.30–1.41 (m, 8H, CH_2_), 1.62–1.71 (m, 4H, CH_2_), 3.67 (t, *J* = 7.5 Hz, 2H, H-1), 4.42 (dt, *J*_H-8,F_ = 47.5 Hz, *J*_H-8,H-7_ = 6.5 Hz, 2H, H-8), 7.70–7.71 (m, 2H, Ar), 7.83–7.84 (m, 2H, Ar); ^13^C-NMR (125 MHz, CDCl_3_): δ 25.04 (d, *J* = 5.5 Hz, CH_2_), 26.73 (d, *J* = 4.5 Hz, CH_2_), 28.52 (CH_2_), 28.82 (d, *J* = 33.2 Hz, CH_2_, C-6), 29.03 (d, *J* = 4.1 Hz, CH_2_, C-5), 30.32 (d, *J* = 19.4 Hz, CH_2_, C-7), 37.98 (CH_2_), 84.15 (d, *J* = 163.9 Hz, CH_2_F) , 123.14 (CH, arom), 132.16 (C, arom), 133.83 (CH, arom), 168.46 (C, CO_amide_). ^19^F-NMR (564 MHz, CDCl_3_): δ −218.04 (tt, *J*_F,H-8_ = 47.5, *J*_F,H-7_ = 24.9 Hz).

##### Route 2 *via fluorination with KF and a phase-transfer catalyst*

A mixture of KF (20 mg, 0.35 mmol, 5 eq) and K_2_CO_3_ (3.5 mg, 0.025 mmol, 0.4 eq) in CH_3_CN (1 mL) was stirred at RT. This was followed by addition of a solution of Kryptofix [2.2.2] (30 mg, 0.08 mmol, 1.1 eq) in CH_3_CN (0.5 mL), which was previously dried twice using co-distillation in CH_3_CN (1 mL) under reduced pressure. After addition of the solution of compound **6** (30 mg, 0.07 mmol, 1 eq) in CH_3_CN (0.5 mL) which was previously dried twice using CH_3_CN (1 mL) through co-distillation under reduced pressure, the mixture was heated under reflux (100 °C) for 30 min. TLC (EtOAc–*n*-hexane = 2:8) analysis indicated formation of the product **4** (R*_f_* = 0.50). A portion of CH_3_CN (5 mL) was added, and the mixture was sequentially filtered through an Al cartridge and RC 18 cartridge. The filtrate was concentrated at 40 °C under reduced pressure to afford a yellow colored crude residue (65 mg). This residue was purified by flash chromatography using a 1.5 cm diameter column with 10 g of silica gel under a gradient eluent condition of EtOAc–*n*-hexane = 1:9→EtOAc–*n*-hexane = 2:8. Product **4** was obtained as a white solid in 62% yield (12 mg). After recrystallization from CH_3_OH, a colorless crystal was obtained in 40% yield (8 mg). m.p. 51–52 °C. The spectroscopic data including ^1^H- and ^19^F-NMR and HRMS were consistent with the data obtained using route 1. Elemental analysis for C_16_H_20_FNO_2_: Calcd. C, 69.29; H, 7.27; N, 5.05. Found C, 69.27; H, 7.34; N, 5.37.

#### *8-Fluorooctan-1-amine* (**5**)

Pattison *et al.* previously reported a method for the preparation of compound **5** [34,35]. A mixture of compound **4** (300 mg, 1.08 mmol) and EtOH (1 mL) in a two-necked round bottomed flask (25 mL) was heated at 80 °C. To this mixture, NH_2_NH_2_·H_2_O (162 μL, 64 wt %, 3.24 mmol, 3 eq) was added. The colorless solution was then stirred for 4 h. TLC analysis was performed using two types of eluting conditions and indicated the consumption of the starting material **4** (R*_f_* = 0.62, EtOAc–*n*-hexane = 4:6) and the formation of product **5** (R*_f_* = 0.22, CH_3_OH:CHCl_3_:NH_3_ = 50%:49%:1%). An anionic exchange resin (OH^−^) was added to the mixture which was then filtered by gravity filtration. The filtrate was concentrated under reduced pressure at 40 °C to afford a dark brown oil (200 mg) with a strong odor. The residue was purified using flash chromatography in a 2.5 cm-diameter column with 30 g of silica gel using CH_3_OH:CHCl_3_:Et_3_N = 50%:49%:1% as an eluent to afford the product **5** as a yellowish oil in 60% yield (95 mg) Pattison *et al.* reported an 81% yield from the hydrogenation of 8-fluorooctane nitrile using LiAlH_4_; a 56% yield was reported by Windhorst *et al.* from the hydrogenation of 7-fluoroheptane nitrile using NaBH_4_. Calcd. C_8_H_18_FN, MW = 147.2, ESI + Q-TOF MS, M = 147.1 (*m*/*z*), [M + H]^+^ = 148.1; HRMS-ESI , Calcd. [M + H]^+^ = 148.1502; found: [M + H]^+^ = 148.1497; Elemental analysis: Calcd. C, 65.26; H, 12.32; N, 9.51. Found C, 65.16; H, 12.16; N, 9.68. ^1^H-NMR (500 MHz, CD_3_OD): δ 1.34–1.40 (m, 8H, CH_2_), 1.47–1.49 (m, 2H, CH_2_), 1.62–1.69 (m, 2H, CH_2_), 2.65 (bs, 2H, CH_2_NH_2_), 4.39 (dt, *J*_H-8,F_ = 47.5 Hz, *J*_H-8,H-7_ = 6 Hz, 2H, H-8); ^13^C-NMR (125 MHz, CD_3_OD): δ 26.27 (CH_2_), 26.31 (CH_2_), 27.87 (CH_2_), 30.38 (d, *J* = 21.2 Hz, CH_2_), 31.54 (d, *J* = 20.0 Hz, CH_2_), 33.26 (CH_2_), 42.34 (CH_2_), 84.88 (d, *J* = 163.4 Hz, CH_2_F). ^19^F-NMR (470 MHz, CD_3_OD): δ −218.44 (tt, *J*_F,H-8_ = 47.5, *J*_F,H-7_ = 24.5 Hz).

#### *8-Fluorooctyl Fenbufen Amide* (FOFA, **1**)

A mixture of HBTU (68 mg, 0.17 mmol, 1.2 eq), fenbufen (68 mg, 0.15 mmol, 1 eq) and diisopropyl-ethylamine (49.5 μL, 0.3 mmol, 2 eq) in DMF (0.5 mL) was stirred at RT for 5 min. Compound **5** (24 mg, 0.16 mmol, 1.1 eq) was then added to this mixture and the reaction was allowed to stir for another 5 min. TLC (acetone–*n*-hexane = 5:5) analysis indicated the consumption of fenbufen (R*_f_* = 0.33) and formation of the intermediate ester (R*_f_* = 0.65). Finally, the product FOFA **1** (R*_f_* = 0.38) was observed through TLC analysis using a developing solvent of acetone–*n*-hexane = 3: 7. The mixture was then partitioned between saturated aqueous NaHCO_3(aq)_ (10 mL × 2) and CH_2_Cl_2_ (20 mL) in a separation funnel. The organic layer was dried using Na_2_SO_4_ followed by filtration through a celite pad. The filtrate was concentrated under reduced pressure to give a dark brown viscous oil (100 mg). The residue was purified by flash chromatography using a 2.5 cm-diameter column with 20 g of silica gel under eluting conditions using acetone–CHCl_3_ = 1:49 to give the product FOFA (**1**) as a white solid in 75% yield (45 mg). After recrystallization from CH_3_CH_2_OH, off-white needle-shaped crystals were obtained in 67% yield (39 mg). m.p.126–128 °C. Calcd. C_24_H_30_FNO_2_, MW = 383.5, ESI + Q-TOF MS, M = 383.2 (*m*/*z*), [M + H]^+^ = 384. 3, [M + Na]^+^ = 406.2; HRMS-ESI, Calcd. [M + H]^+^ = 384.2339, [M + Na]^+^ = 406.2158, [M + K]^+^ = 422.1898; found: [M + H]^+^ = 384.2327, [M + Na]^+^ = 406.2147, [M + K]^+^ = 422.1883; Elemental analysis: Calcd. C, 75.16; H, 7.88; N, 3.65. Found C, 75.28; H, 7.74; N, 3.77. ^1^H-NMR (500 MHz, CDCl_3_): δ 1.35–1.39 (m, 8H, CH_2_), 1.49–1.52 (m, 2H, CH_2_), 1.63–1.71 (m, 2H, CH_2_), 2.63 (t, *J* = 6.5 Hz, 2H, CH_2_), 3.23–3.27 (m, 2H, CH_2_), 3.40–3.41 (m, 2H, CH_2_), 4.42 (dt, *J*_H-8,F_ = 47.5 Hz, *J*_H-8,H-7_ = 6.0 Hz, 2H, H-8), 5.78 (bs, 1H, CONH), 7.39–7.41 (m, 1H, Ar), 7.46–7.49 (m, 2H, Ar), 7.63 (d, *J* = 7.0 Hz, 2H, Ar), 7.69 (d, *J* = 9.0 Hz, 2H, Ar), 8.06 (d, *J* = 8.5 Hz, 2H, Ar); ^13^C-NMR (125 MHz, CDCl_3_): δ 25.04 (CH_2_), 25.08 (CH_2_), 26.75 (CH_2_), 29.09 (CH_2_), 29.12 (CH_2_), 29.57 (CH_2_), 30.30 (d, *J* = 20.7 Hz, CH_2_), 34.23 (CH_2_), 39.61 (CH_2_), 84.16 (d, *J* = 163.9 Hz, CH_2_F), 127.25 (CH, arom), 128.24 (CH, arom), 128.66 (CH, arom), 128.94 (CH, arom), 135.26 (C, arom), 139.82 (C, arom), 145.93 (C, arom), 171.97 (C, CO_amide_), 198.76 (C, C=O). ^19^F-NMR (470 MHz, CDCl_3_): δ −218.02 (tt, *J*_F,H-8_ = 47.5, *J*_F,H-7_ = 24.9 Hz).

### 3.3. Radiochemistry

#### Preparation of *8-[^18^F]fluorooctyl-1-amine* ([^18^F]F**-5**)

A reaction vessel containing 315 mCi of K[^18^F]F was obtained from enriched [18O]H_2_O via proton irradiation followed by elution through a column of AGI X 8 resin. A solution of Kryprtofix[2.2.2] (20 mg) in CH_3_CN (1 mL) was added to the vessel followed by heating at 100 °C under fumes of He gas according to a previously described procedure [26]. The formed Kryptofix-^+^K-[^18^F]^−^F complex was dried via repeated dissolution and distillation. A mixture of tosylate **6** (20 mg) and *t*-BuOH (0.4 mL) in CH_3_CN (1 mL) was added to this reaction vessel. The reaction was allowed to stir at 100 °C and a pressure of 250 kpa for 10 min. After cooling to 90 °C, the mixture was purged with He fumes for 2 min and was concentrated under reduced pressure for 2 min. In a probe analysis, the obtained intermediate, [^18^F]F**-4**, was purified using normal phase HPLC as stated in the general section with a retention time (*t*_R_) of 13.4 min. Upon cooling the sample down to 30 °C, a mixture of aqueous H_2_NNH_2_·H_2_O (40 μL) and EtOH (2 mL) was injected. The reaction was then carried out at 70 °C for 15 min and was subsequently cooled to 30 °C. The mixture was eluted through a set up comprising two alumina cartridges and an RC-18 cartridge. The eluents were filtered through a 0.45 μM nylon filter and were then washed with EtOH (1.5 mL). The filtrates were combined to give crude 8-[^18^F]fluorooctyl-1-amine [^18^F]F**-5** in 60% yield (131.2 mCi). Further purification using either normal or reverse phase HPLC was unsuccessful. Thus purification was peformed at the next stage of conjugation with fenbufen. The mixture (3 mL) was transferred to a round bottomed flask (10 mL) and was concentrated under under reduced pressure (20 mbar) at 40 °C for 15 min to afford [^18^F]F**-5** with a radioactivity of 14 mCi; this compound was then used for the next conjugation experiment.

#### Preparation of 8*-[^18^F]fluorooctylfenbufen amide* ([^18^F]F**-1**)

A mixture of fenbufen (20 mg, 0.078 mmol, 1 eq), HBTU (38 mg, 0.1 mmol, 1.3 eq), diisopropyl ethylamine (26 μL, 0.157 mmol, 2 eq) and DMF (150 μL) in a microcentrifuge tube was sonicated for 5 min. A brown solution of activated fenbufen ester was formed and was added to the flask containing [^18^F]F**-5**. The reaction mixture was stirred at RT for 15 min. The mixture was then purified through a set up comprising an Al cartridge, an RC-18 cartridge, and a 0.45 μm Nylon Millipore filter. The filtrates, along with washings of CH_2_Cl_2_ (5 mL), were combined and concentrated under reduced pressure at 50 °C to give the crude product [^18^F]F**-1** with a radioactivity of 4.82 mCi. Followed by addition of EtOH (0.5 mL), the mixture was purified by RP-HPLC under eluting condition of H_2_O:CH_3_CN = 30:70 in isocratic mode, as described in the general section. The desired fraction (*t_R_* = 15.4 min) was isolated and concentrated. After dissolving the sample in CH_3_CH_2_OH (200 μL) and saline (800 μL), the solution was shown to have a radioactivity of 2.81 mCi and was used for the PET imaging study.

### 3.4. Cell Culture for C6 Glioma and Fibroblasts

The procedure was performed as described previously [26] A rat glioma cell line C6 was obtained from the American Type Cell Collection (ATCC, Manassas, VA, USA). The C6 glioma cells were cultured with Dulbecco’s modified Eagles medium (DMEM) containing 10% fetal calf serum (Gibco, Thermo Fisher Scientific, MA, USA) under 5% of CO_2_ at 37 °C in a 96-well microtiterplate. The cells were subcultured upon reaching 80%–90% confluency. The fibroblast cell line, 3T3, was provided by our collaborator, Dr. Ya-Hwei Wu who purchased it from ATCC (American Type Cell Collection). The cells were maintainedin the same culture conditions described for the C6 cells.

### 3.5. Animal Model of Cells Implanted in Rat Brains

Sprague-Dawley (SD) rats (8 weeks of age) were obtained from the BioLasco Animal Co. (Taipei, Taiwan). All studies involving animals were conducted in compliance with federal and institutional guidelines, including proper animal housing sanitation, clean water supply, sufficient feeding, 12-h day/night light control, and humidity/temperature control. The Institutional Animal Care and Use Committee of Chang Gung University approved the animal experiments (grant no. CGU 14-164). Two normal rats were used for the biodistribution experiment. One tumor rat was used for PET study. Three weeks before performing PET imaging study, adult Sprague-Dawley male rats (ages 6–8 weeks, body weight 250–300 g) were stereotactically xenografted with 5 μL (5 × 10^5^) of C6 cells in the right hemisphere under isoflurane anesthesia. (American Type Tissue Collection). The animals were placed on heating pad until they have entirely recovered. The animals were transferred to the animal facility. The research staffs monitored the animal body weight, water intake, signs of pain or distress every morning before the PET imaging. If the animals appear lethargic, do not appear to be eating or drinking over 24 h, or weight loss greater than 20% body weight, euthanasia will be carried out to avoid further suffering. The animals were monitored regularly with care in respect with the feeding quality, interaction, and symptom of dystrophy. The tumor growth was monitored by IVIS at the first and second week after implantation procedure. MRI was then used to monitor the tumor size once before the PET imaging study. The tumor can usually grow with a size of 3 mm–5 mm in diameter two to three weeks after implantation. Prior to PET imaging, all rats were affixed with venous and arterial catheters.

### 3.6. MTT Assay (Cell Viability Assay)

The MTT assay was performed as described previously [27]. Cell toxicity was assessed by observing the reducing potential of mitochondria organelle in the cells upon addition of the MTT reagent (3-(4,5-dimethyl-thiazol-2-yl)-2,5-diphenyl tetrazolium bromide). In brief, C-6 glioma cells and fibroblast cells were plated in a 96-well plate (5000/well) and incubated with MEM containing 10% fetal calf serum (Gibco) at 37 °C under a 5% CO_2_ atmosphere, followed by treatment of FOFA. After a 48-h culture period, 50 μL of MTT (5 mg/mL) was added to each well and the cells were incubated for an additional 4 h. The supernatant was removed and 100 μL of DMSO was added to each well to dissolve the formazan crystals formed. The optical absorbance at 570 nm was read by a spectrophotometer.

### 3.7. Analysis of the Stability of [^18^F]FOFA {[^18^F]F-**1**} in Plasma

Before carrying out the *in vivo* imaging experiment, the *ex vivo* stability of [^18^F]F-**1** was assessed using HPLC analysis of the mixture containing [^18^F]F-**1** in blood plasma according to a reported procedure [36]. An aliquot (10 µL, 50 uCi) drawn from the solution containing the [^18^F]F-**1** radiotracer in EtOH (100 uCi/500 uL) was mixed with a volume of 0.2 mL of heparin-pretreated plasma taken from the SD rats (5 IU/mL). After standing for 10 s, 2 min, 10 min and 30 min, an aliquot (20 μL) of the plasma mixture was added to a tube containing a solution of CH_3_CN and H_2_O (7:1, 180 μL). After centrifugation (6500 rpm/200 *g*, Qik Spin, Narellan NSW, Australia) for 1 min, a volume of 100 µL was drawn from the supernatant and was filtered through a membrane filter (0.45 µM, PTFE, Millipore). The filtrate was chromatographed using a reverse phase HPLC system equipped with a semi-preparative column. The elution condition was the same as that described for the radiochemical purification process. The generated radioactivity chromatogram was analyzed to establish a temporal plot for the concentration of the radioactive product and its metabolites.

### 3.8. Biodistribution of [^18^F]F-**1** in Normal Rats

The experimental procedure was similar to that used previously [26]. In brief, [^18^F]FOFA {[^18^F]-**1**} dissolved in 20% EtOH (aq, 0.3 mL) with an activity of approximately 1 mCi was injected into two rats via the tail vein. The two rats were sacrificed subsequently after 2 and 60 min, respectively, to excise various specimens. These included twelve organ tissues such as the brain, liver, spleen, heart, kidney, lung, colon, small bowel, stomach, testes, skull, and muscle. These specimens were submitted for quantification of radioactivity using a solid scintillation gamma counter (Packard 5000, Packard Instrument Co. Laboratory, Meriden, CT, USA). The counting value of each specimen was further divided by the sample weight to give the final expression as a percentage of the injection dose per sample weight (%ID/g).

### 3.9. PET/CT Imaging Study Using [^18^F]F-**1** in an Animal Model

PET scanning experiments were performed within 72 h of the MRI experiments that were used to confirm successful inoculation of the tumors by administering [^18^F]F-**1** via tail vein injections [27]. NanoPET/CT in static mode was employed over 60 min scanning sessions. Data were acquired in list-mode. The raw data within each frame were then binned into three-dimensional sonograms with a span of three and ring difference of 47. The data were corrected for scattering and attenuation using a two-dimensional ordered-subsets expectation-maximization algorithm, which had 16 subsets and four iterations. The sonograms were reconstructed into tomographic images (128 × 128 × 95) with voxel sizes of 0.095 × 0.095 × 0.08 cm^3^.

## Figures and Tables

**Table 1:** Comparison of radiochemical yield (RCY) using different reagent combinations.

Entry	Reagent Combination	RCY	Experiment No.
1	TsCl/pyridine	33% ± 4%	4
2	TsCl/DMAP	39% ± 4%	3
3	Ts_2_O/DMAP	53% ± 1%	4

## Supplementary Materials

Supplementary materials can be accessed at: http://www.mdpi.com/1420-3049/21/3/387/s1.
