# Supplementary material for: Synthesis and Biological Evaluation of an 18Fluorine-Labeled COX Inhibitor—[18F]Fluorooctyl Fenbufen Amide—For Imaging of Brain Tumors
Source: Molecules. 2016 Mar 21;21(3):387. doi: 10.3390/molecules21030387 (PMC6273898; doi:10.3390/molecules21030387)
Supplement: Supplementary file 1 [file molecules-21-00387-s001.pdf]

Supplementary Materials: Synthesis and Biological Evaluation of a Fluorine-18-Labeled COX Inhibitor [18F]Fluorooctyl Fenbufen Amide for Imaging of Brain Tumors

Ying-Cheng Huang, Yu-Chia Chang, Chun-Nan Yeh and Chung-Shan Yu

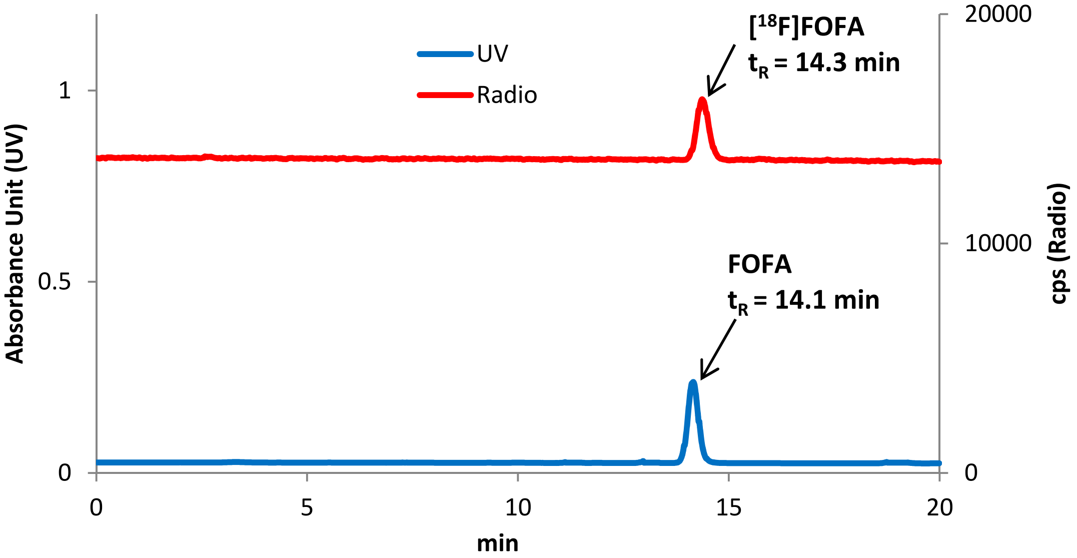

Figure S1. The HPLC chromatogram of the purified [^{18}F]FOFA{[^{18}F]-**1**} along with the authentic sample FOFA **1** for purity assessment using semipreparative RP-HPLC. The injection contained a radioactivity of 40 uCi mixed with a concentration of 0.02 mg/0.2 mL of FOFA **1**.

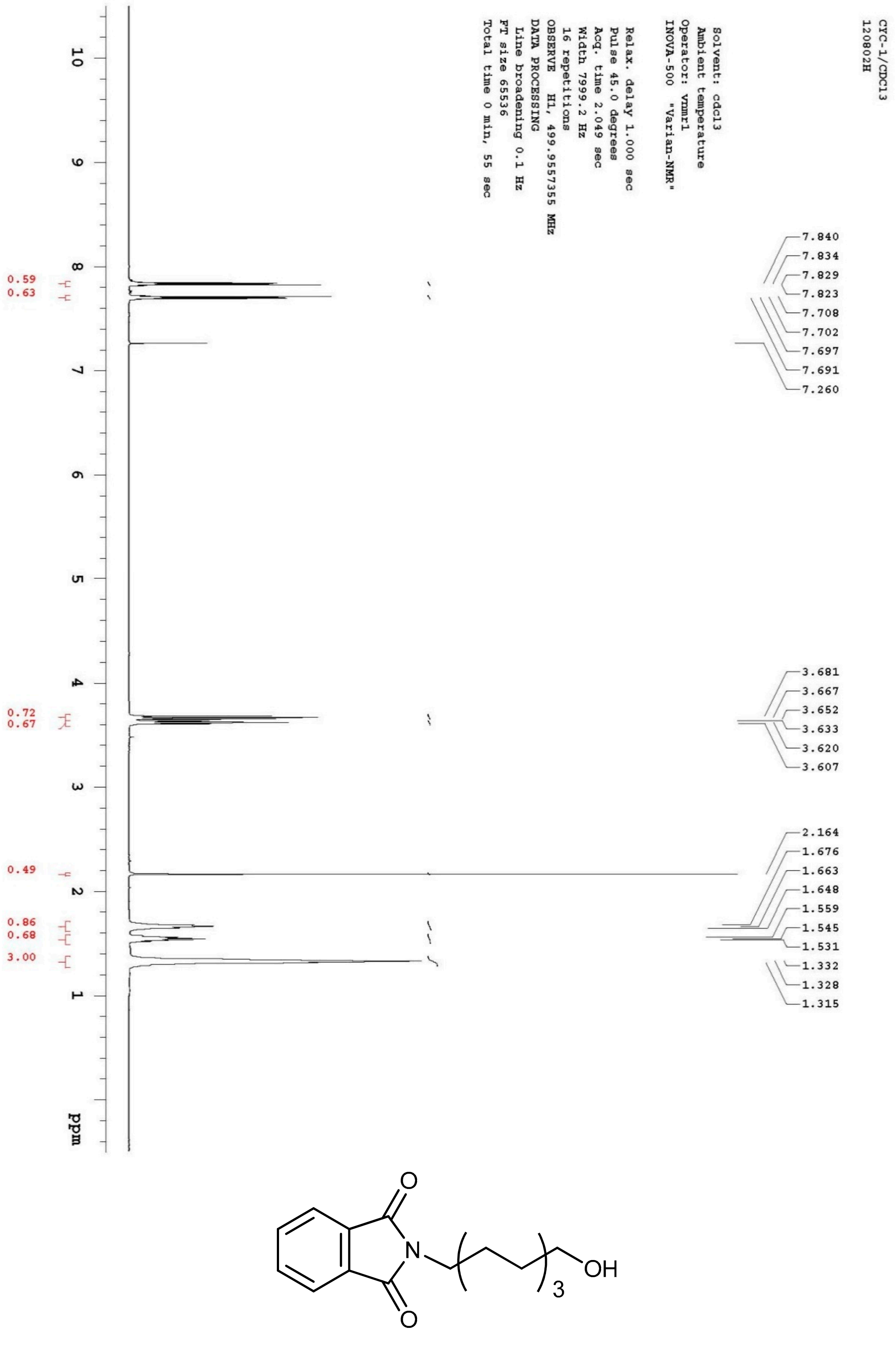

3

Figure S2. ^{1}H-NMR of 2-(8-Hydroxyoctyl)isoindoline-1,3-dione **3**.

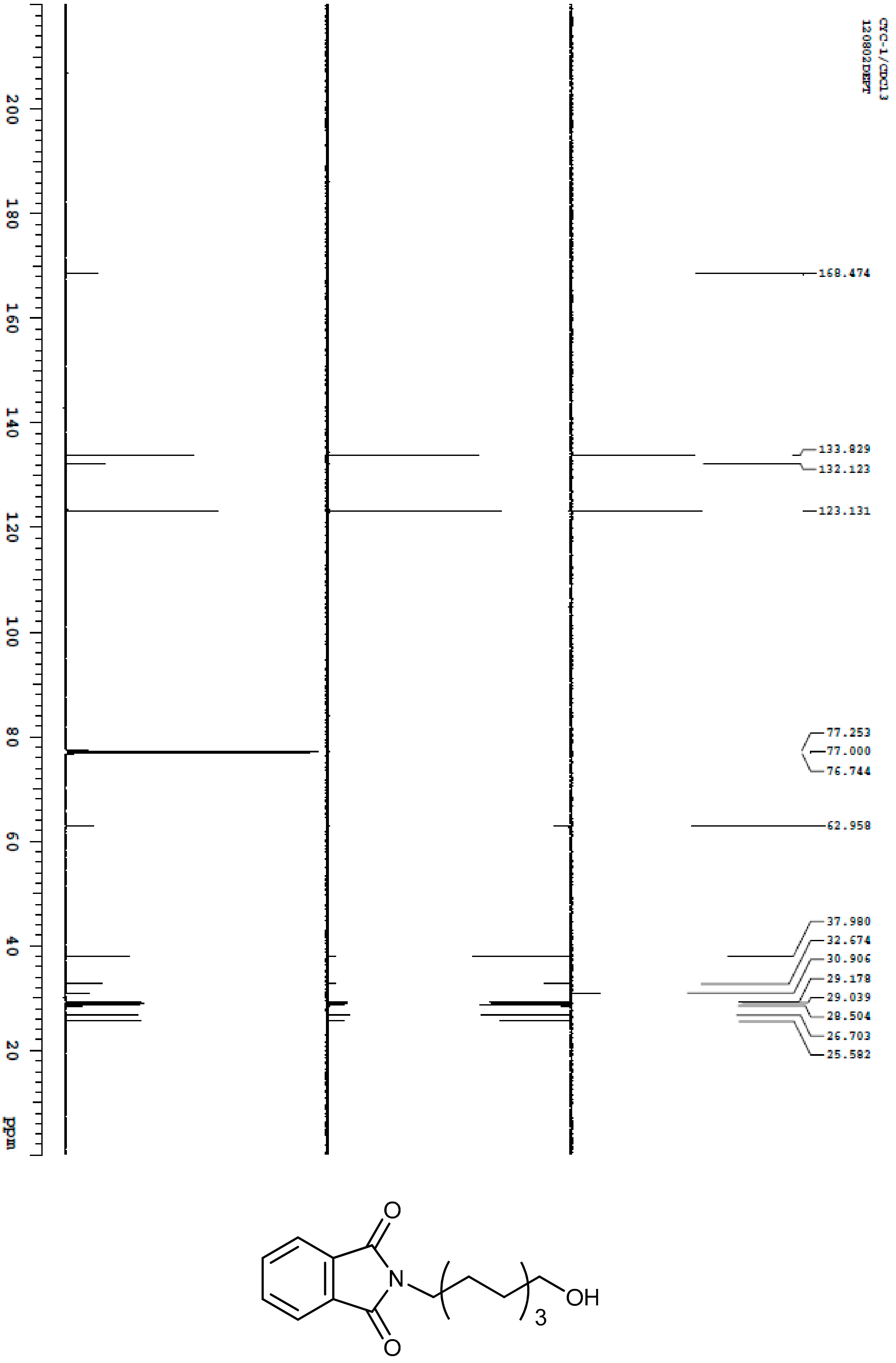

3

Figure S3. ^{13}C-DEPT of 2-(8-Hydroxyoctyl)isoindoline-1,3-dione **3**.

國立交通大學應用化學系

元素分析儀Heraeus CHN-O Rapid 服務報告

姓名: 張育嘉　　　　單位: 清大醫環俞鐘山LAB

收件日: 102.6.25　　　　完成日: 102.6.26

實測值:

樣品名	CYC-1	CYC-1						
N%	5.36	5.44						
C%	69.46	69.48						
H%	7.81	8.11						

樣品名								
N%								
C%								
H%								

推測值:

樣品名	CYC-1							
N%	5.09							
C%	69.79							
H%	7.69							

所使用之標準品: Acetanilide

	理論值	測出值
N%	10.36	10.33
C%	71.09	70.89
H%	6.71	6.66

備註:

儀器負責人簽章: 謝有容　　　　技術員簽章: 技士李蘊明

Figure S4. Elemental analysis of 2-(8-Hydroxyoctyl)isoindoline-1,3-dione **3**.

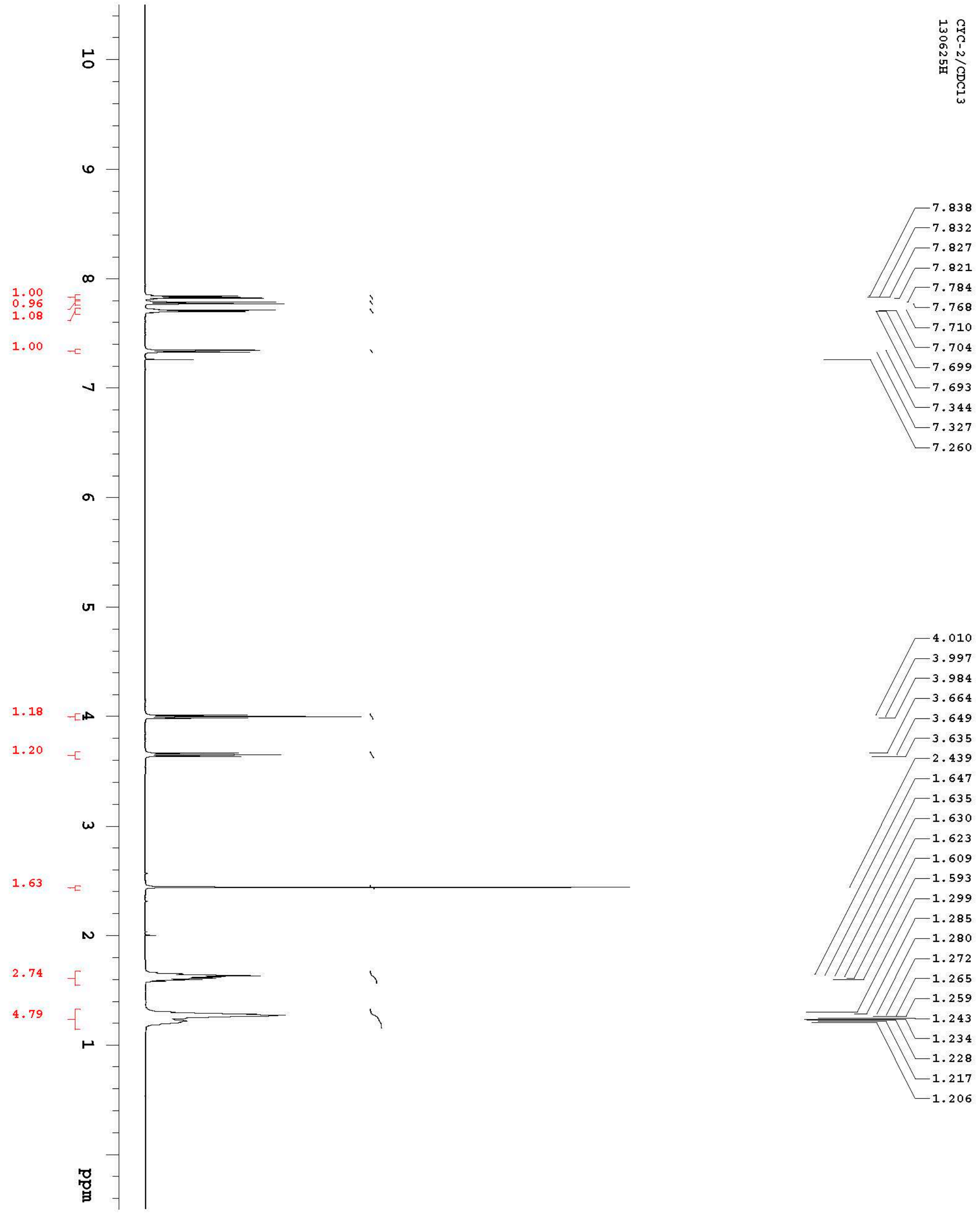

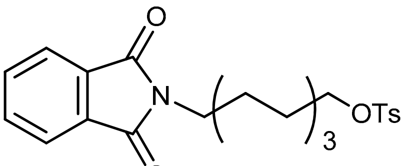

6

Figure S5. ^{1}H-NMR of 8-(1,3-Dioxoisoindolin-2-yl)octyl 4-methylbenzenesulfonate **6**.

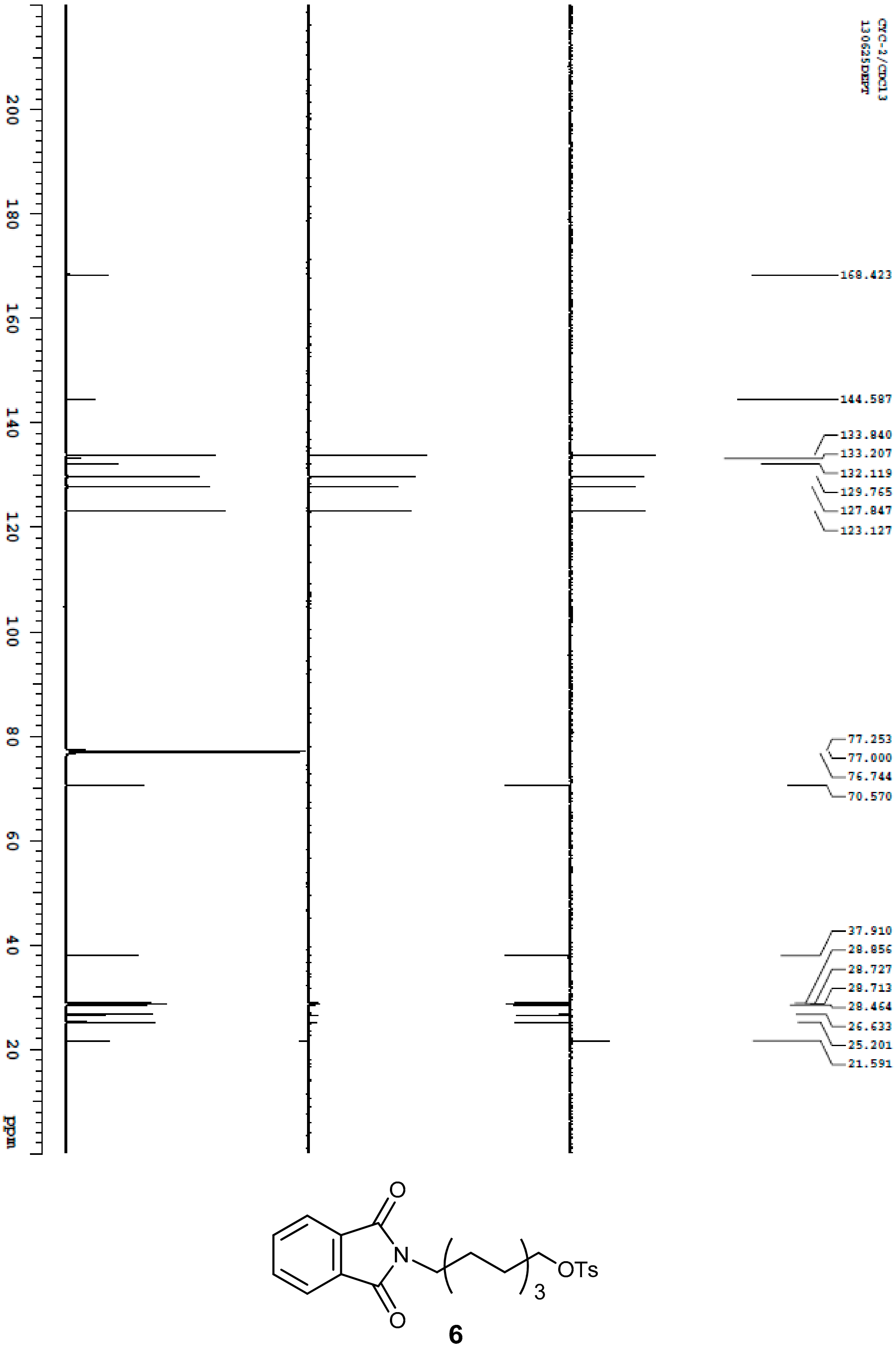

6

Figure S6. ^{13}C-DEPT of 8-(1,3-Dioxoisoindolin-2-yl)octyl 4-methylbenzenesulfonate **6**.

國立交通大學應用化學系

元素分析儀Heraeus CHN-O Rapid 服務報告

姓名: 張育嘉 單位: 清大醫環俞鐘山LAB

收件日: 102.6.25 完成日: 102.6.26

實測值:

樣品名	CYC-2	CYC-2						
N%	3.40	3.51						
C%	64.32	64.24						
H%	6.33	5.99						

樣品名								
N%								
C%								
H%								

推測值:

樣品名	CYC-2							
N%	3.26							
C%	64.31							
H%	6.34							

所使用之標準品: Acetanilide

	理論值	測出值
N%	10.36	10.33
C%	71.09	70.89
H%	6.71	6.66

備註:

儀器負責人簽章: [illegible] 技術員簽章: 技士李蘊明

Figure S7. Elemental analysis of 8-(1,3-Dioxoisoindolin-2-yl)octyl 4-methylbenzenesulfonate **6**.

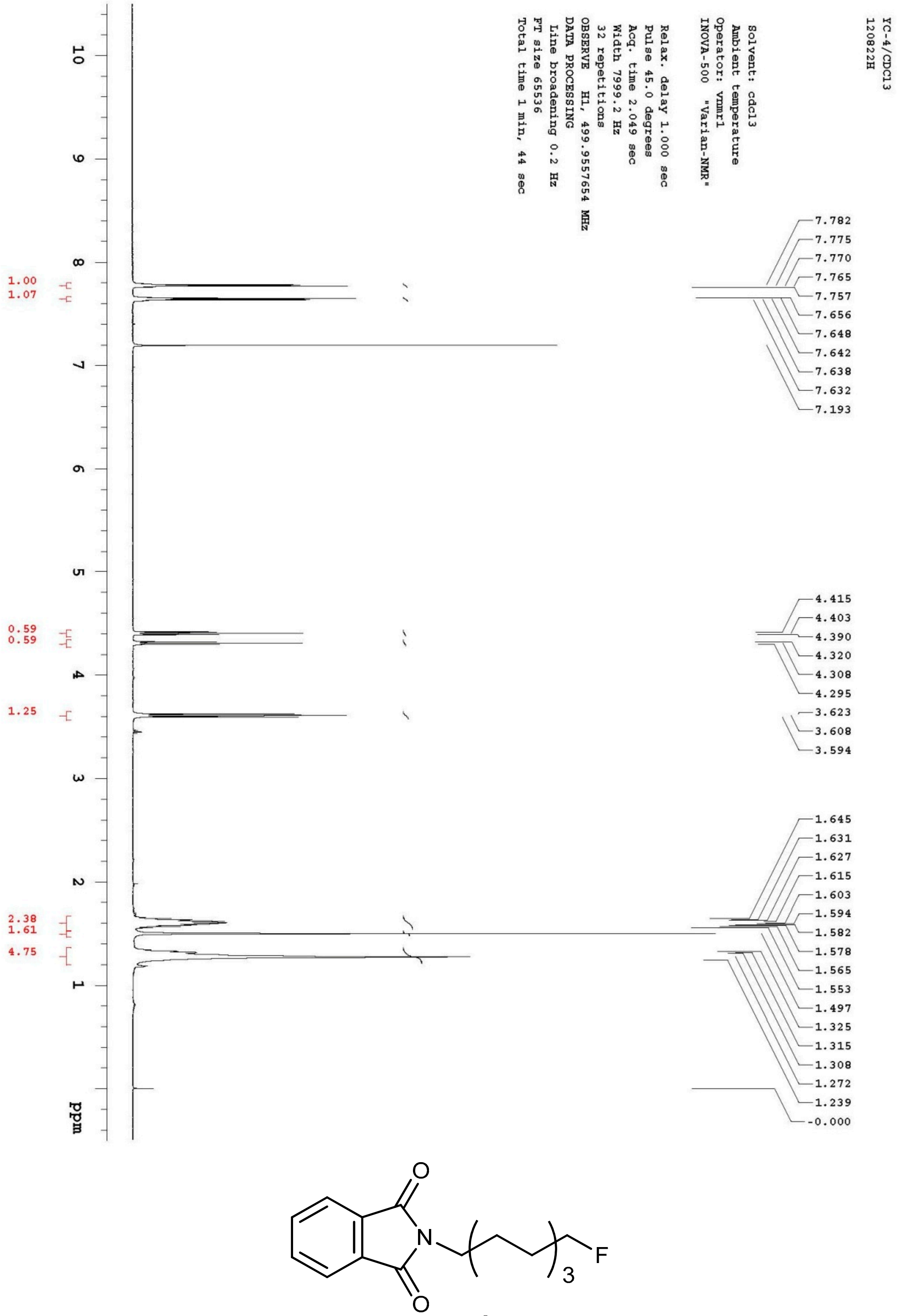

4

Figure S8. ^{1}H-NMR of 2-(8-Fluorooctyl)isoindoline-1,3-dione **4**.

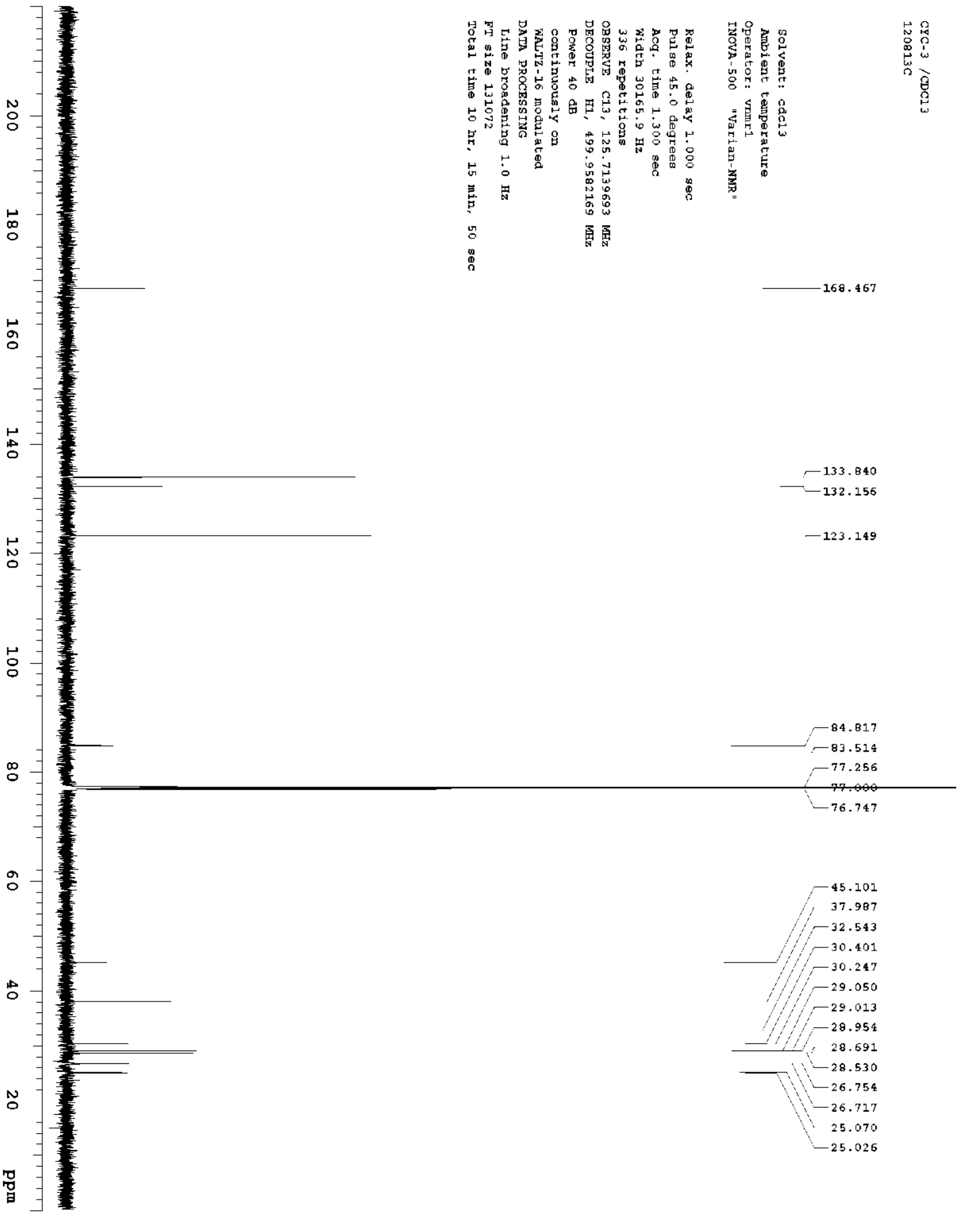

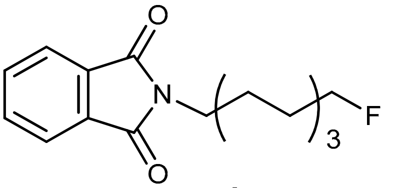

4

Figure S9. ^{13}C-NMR of 2-(8-Fluorooctyl)isoindoline-1,3-dione **4**.

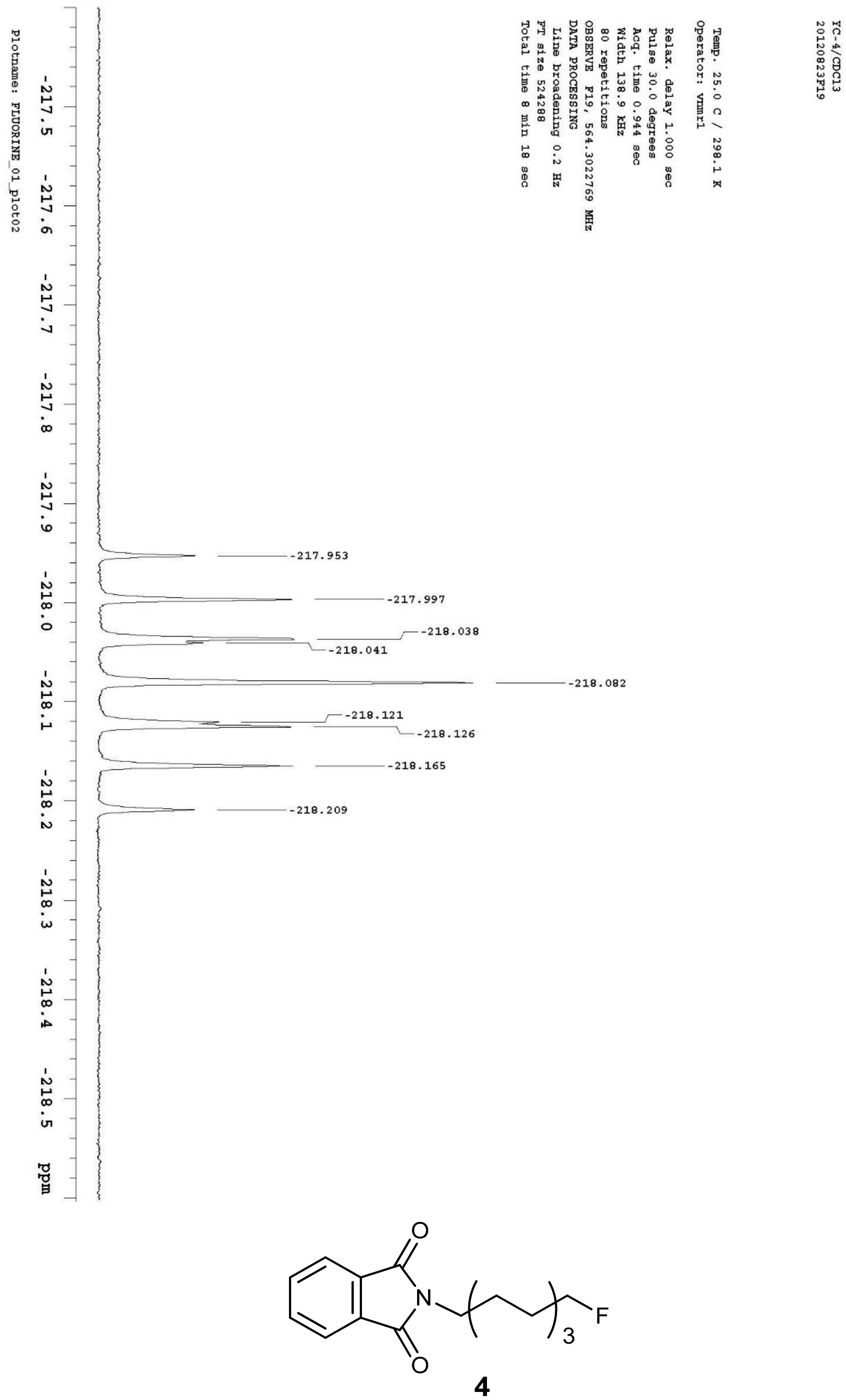

4

Figure S10. ^{19}F-NMR of 2-(8-Fluorooctyl)isoindoline-1,3-dione **4**.

國立交通大學應用化學系

元素分析儀 Heraeus CHN-O Rapid 服務報告

姓名: 張育嘉 單位: 清大醫環俞鐘山 LAB

收件日: 102.6.25 完成日: 102.6.26

實測值:

樣品名	CYC-4	CYC-4						
N%	5.37	5.40						
C%	69.27	69.35						
H%	7.34	7.31						

樣品名								
N%								
C%								
H%								

推測值:

樣品名	CYC-4							
N%	5.05							
C%	69.29							
H%	7.27							

所使用之標準品: Acetanilide

	理論值	測出值
N%	10.36	10.33
C%	71.09	70.89
H%	6.71	6.66

備註:

儀器負責人簽章: 謝有容 技術員簽章: 技士李蘊明

Figure S11. Elemental analysis of 2-(8-Fluorooctyl)isoindoline-1,3-dione **4**.

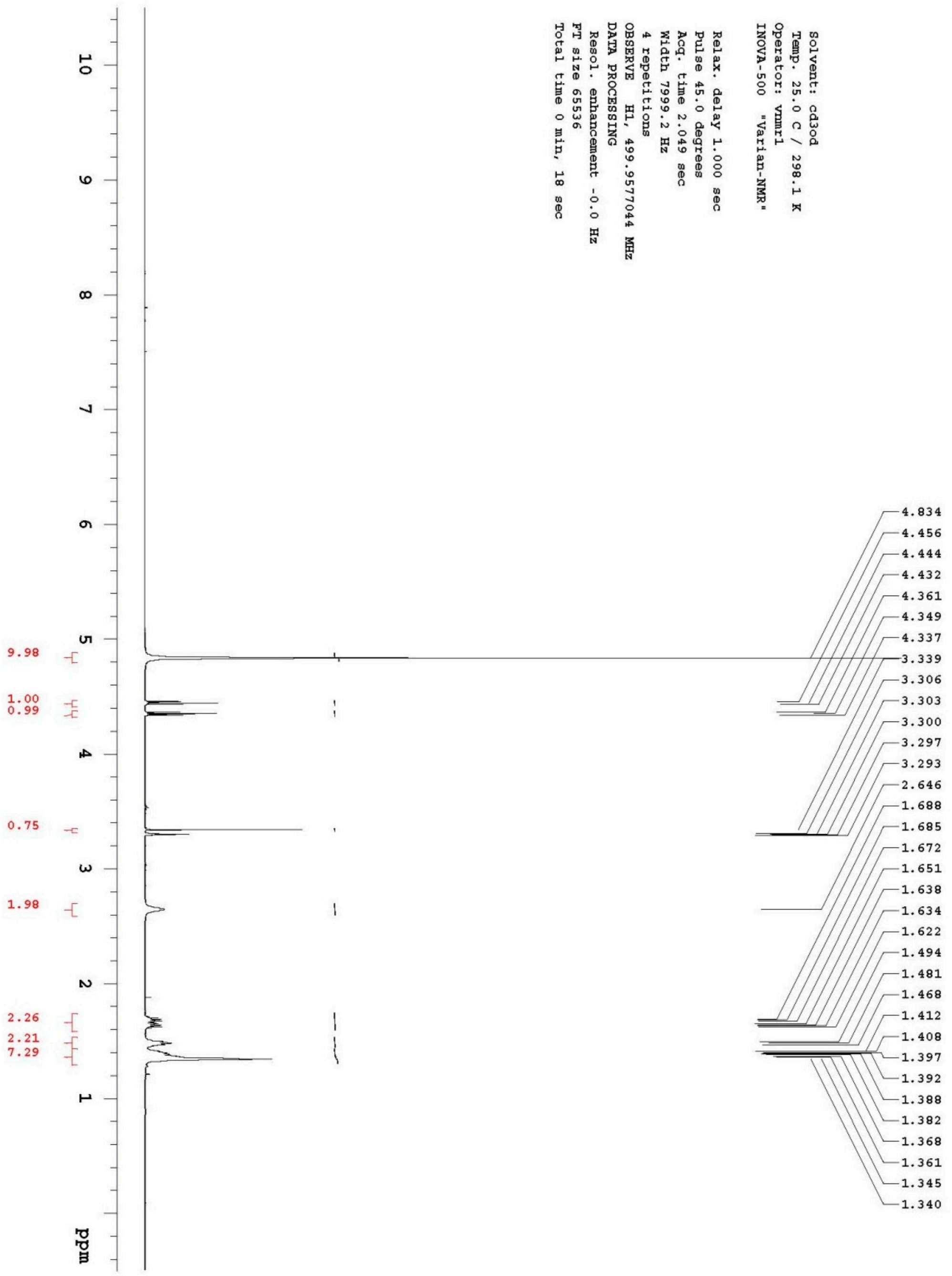

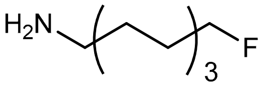

5

Figure S12. ^{1}H-NMR of 8-Fluorooctan-1-amine **5**.

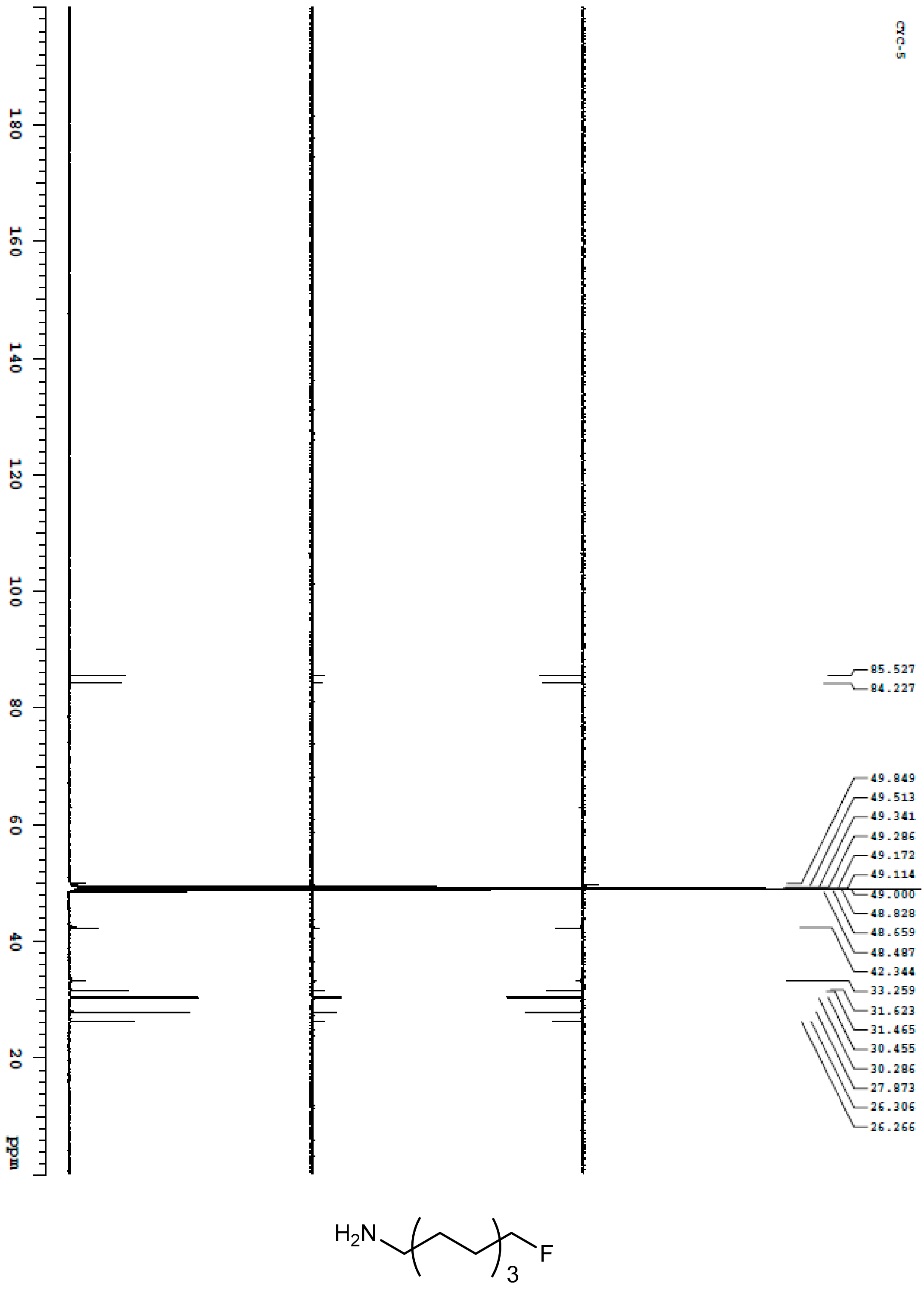

5

Figure S13. ^{13}C-DEPT of 8-Fluorooctan-1-amine **5**.

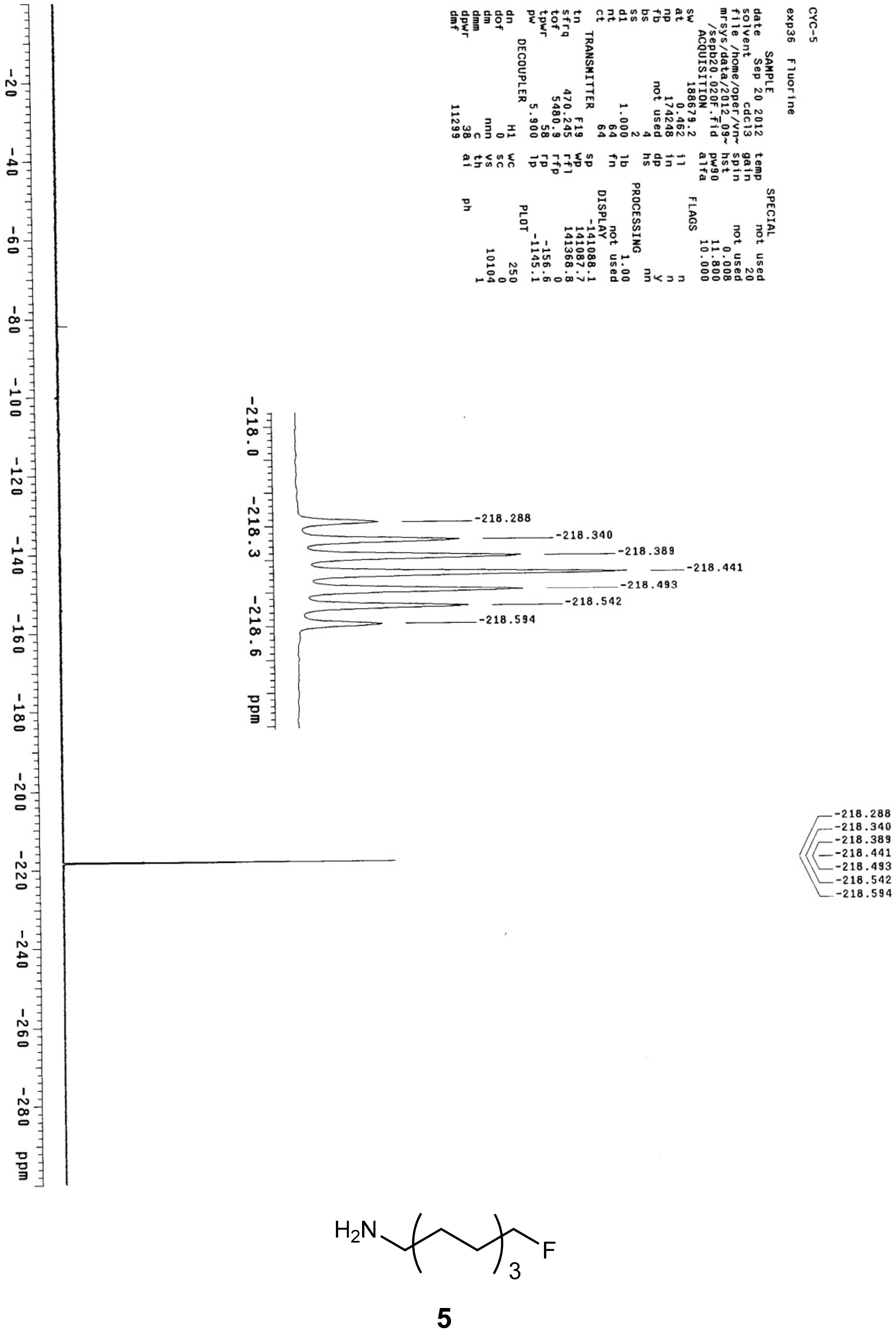

5

Figure S14. ^{19}F-NMR of 8-Fluorooctan-1-amine **5**.

國立交通大學應用化學系

元素分析儀 Heraeus CHN-O Rapid 服務報告

姓名: 張育嘉 單位: 清大醫環俞鐘山 LAB

收件日: 102.6.25 完成日: 102.6.26

實測值:

樣品名	CYC-5	CYC-5						
N%	9.72	9.68						
C%	65.52	65.16						
H%	12.14	12.16						

樣品名								
N%								
C%								
H%								

推測值:

樣品名	CYC-5							
N%	9.51							
C%	65.26							
H%	12.32							

所使用之標準品: Acetanilide

	理論值	測出值
N%	10.36	10.33
C%	71.09	70.89
H%	6.71	6.66

備註:

儀器負責人簽章:

技術員簽章:

Figure S14. Elemental analysis of 8-Fluorooctan-1-amine **5**.

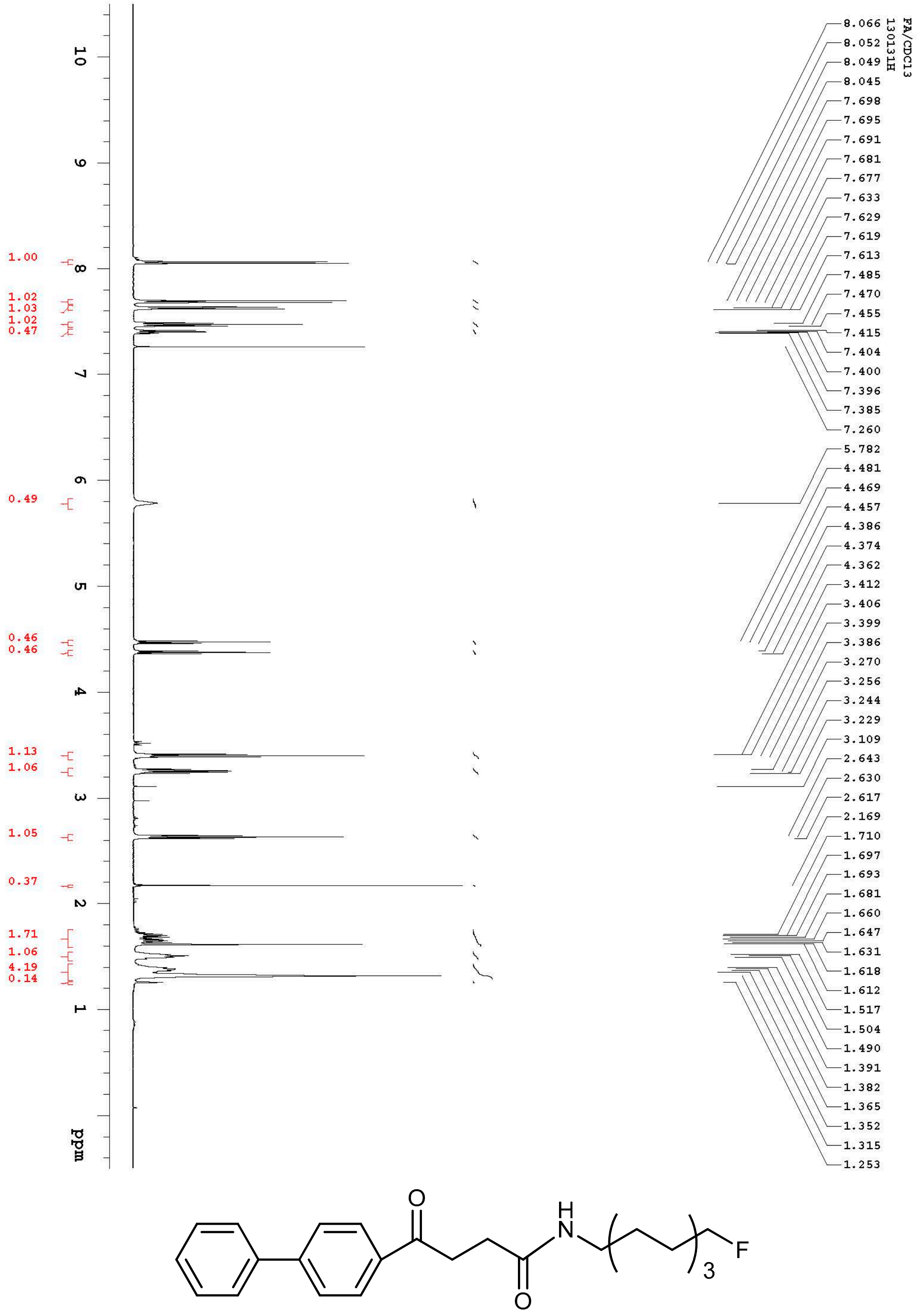

FOFA, 1

Figure S16. ^{1}H-NMR of 8-Fluorooctyl fenbufen amide **1**.

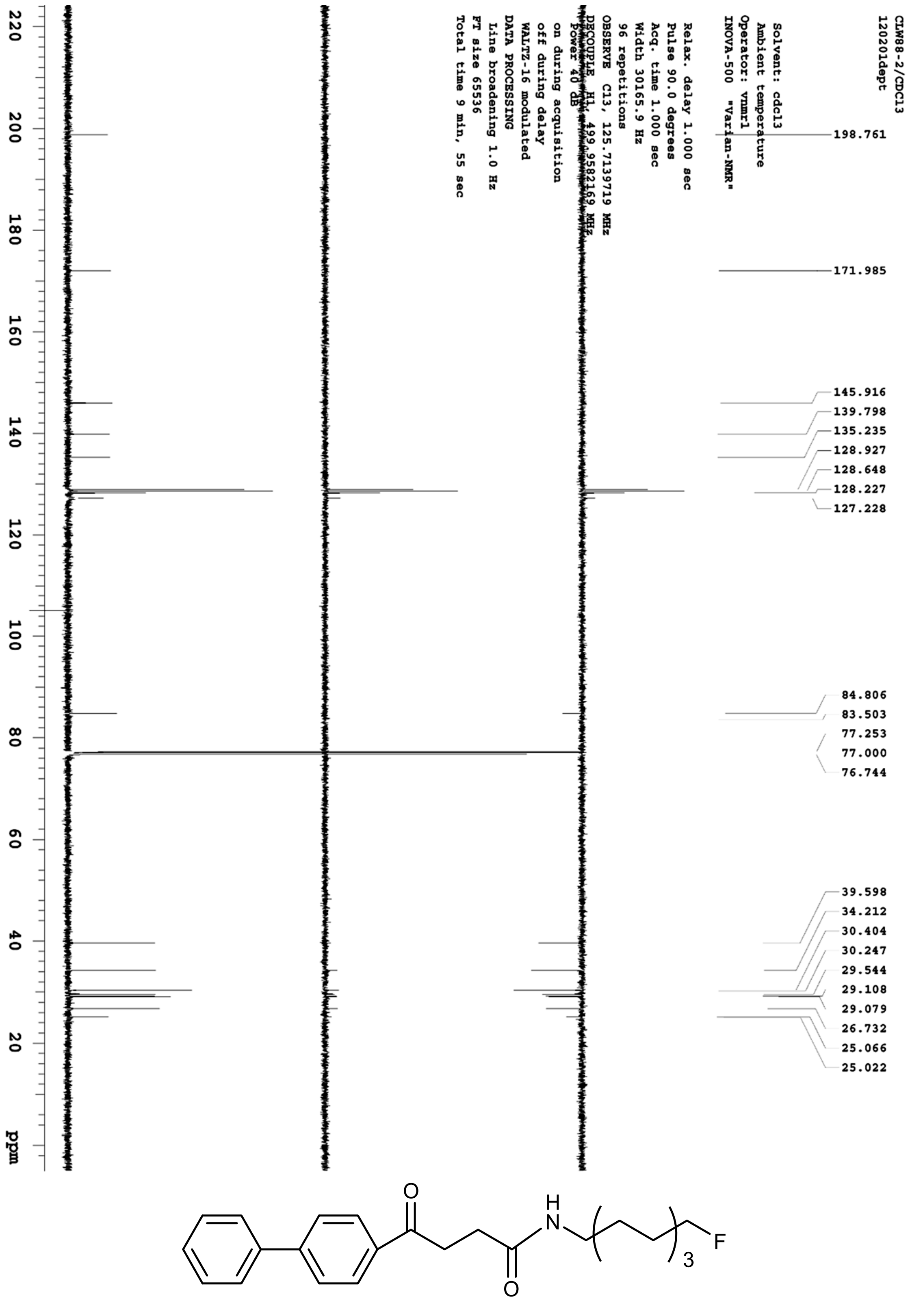

FOFA, 1

Figure S17. ^{13}C-NMR of 8-Fluorooctyl fenbufen amide **1**.

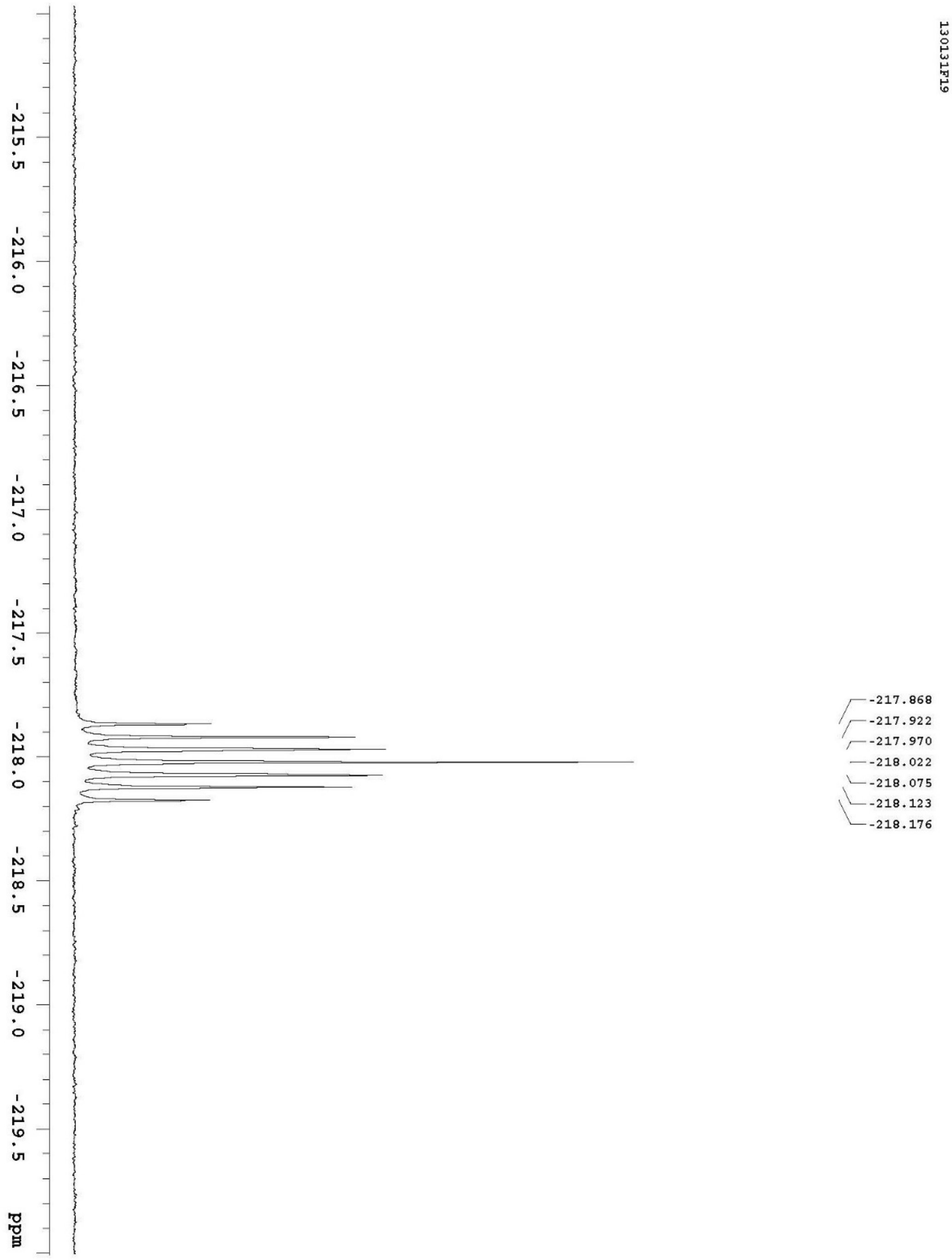

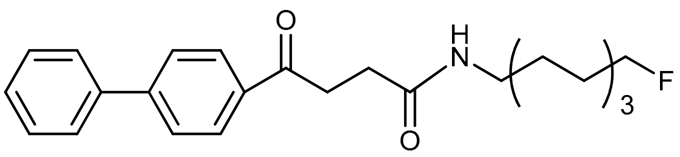

FOFA, 1

Figure S18. ^{19}F-NMR of 8-Fluorooctyl fenbufen amide **1**.

國立交通大學應用化學系

元素分析儀 Heraeus CHN-O Rapid 服務報告

姓名: 張育嘉　　　　單位: 清大醫環俞鐘山 LAB

收件日: 102.6.25　　　　完成日: 102.6.26

實測值:

樣品名	FOFA	FOFA						
N%	3.77	3.41						
C%	75.28	75.04						
H%	7.74	7.52						

樣品名								
N%								
C%								
H%								

推測值:

樣品名	FOFA							
N%	3.65							
C%	75.16							
H%	7.88							

所使用之標準品: **Acetanilide**

	理論值	測出值
N%	10.36	10.33
C%	71.09	70.89
H%	6.71	6.66

備註:

儀器負責人簽章:　　　　技術員簽章:

Figure S19. Elemental analysis of 8-Fluorooctyl fenbufen amide **1**.
